# Role of Subunit Exchange and Electrostatic Interactions on the Chaperone Activity of *Mycobacterium leprae* HSP18

**DOI:** 10.1371/journal.pone.0129734

**Published:** 2015-06-22

**Authors:** Sandip Kumar Nandi, Alok Kumar Panda, Ayon Chakraborty, Sougata Sinha Ray, Ashis Biswas

**Affiliations:** 1 School of Basic Sciences, Indian Institute of Technology Bhubaneswar, Bhubaneswar, India; 2 Wipro GE Healthcare, Kolkata, India; University of Pittsburgh, UNITED STATES

## Abstract

*Mycobacterium leprae* HSP18, a major immunodominant antigen of *M*. *leprae* pathogen, is a small heat shock protein. Previously, we reported that HSP18 is a molecular chaperone that prevents aggregation of different chemically and thermally stressed client proteins and assists refolding of denatured enzyme at normal temperature. We also demonstrated that it can efficiently prevent the thermal killing of *E*. *coli* at higher temperature. However, molecular mechanism behind the chaperone function of HSP18 is still unclear. Therefore, we studied the structure and chaperone function of HSP18 at normal temperature (25°C) as well as at higher temperatures (31–43°C). Our study revealed that the chaperone function of HSP18 is enhanced significantly with increasing temperature. Far- and near-UV CD experiments suggested that its secondary and tertiary structure remain intact in this temperature range (25–43°C). Besides, temperature has no effect on the static oligomeric size of this protein. Subunit exchange study demonstrated that subunits of HSP18 exchange at 25°C with a rate constant of 0.018 min^-1^. Both rate of subunit exchange and chaperone activity of HSP18 is found to increase with rise in temperature. However, the surface hydrophobicity of HSP18 decreases markedly upon heating and has no correlation with its chaperone function in this temperature range. Furthermore, we observed that HSP18 exhibits diminished chaperone function in the presence of NaCl at 25°C. At elevated temperatures, weakening of interactions between HSP18 and stressed client proteins in the presence of NaCl results in greater reduction of its chaperone function. The oligomeric size, rate of subunit exchange and structural stability of HSP18 were also found to decrease when electrostatic interactions were weakened. These results clearly indicated that subunit exchange and electrostatic interactions play a major role in the chaperone function of HSP18.

## Introduction

Exposure to temperatures beyond normal conditions or other stress factors may be lethal to cells. Thus, cells from several organisms have developed stress induced responses in order to guard themselves by the synthesis of a highly conserved set of proteins known as heat shock proteins (HSPs). An Italian scientist R. Ritossa observed the gene expression of puffing in the chromosomes of *Drosophila melanogaster* after exposure to heat which laid the pathway towards the discovery of HSPs [1]. They are upregulated with an acute increase in temperature. Evidences reveal that these HSPs are essential for the survival of cells at both normal and elevated temperatures. Often they play a pivotal role in cellular homeostasis and stress response [2]. Though the location of these proteins varies, mostly they are found in cytosol, nucleus, mitochondria and endoplasmic reticulum. Based on molecular weights, HSPs in archaea [3, 4], human [2], bacteria [5] and plants [5, 6] have been classified into five different categories, one of them is small heat shock protein family.

Small heat shock proteins (sHSPs) are ubiquitously expressed [7]. They exhibit tissue specific expression in a variety of organisms with a molecular weight ~12–43 kDa [8]. Most sHSPs are large molecular weight assemblies that have variable quaternary structure with a dynamic property which enable its subunits to freely exchange with oligomers. sHSPs usually possess a highly conserved "α-crystallin domain" at the centre, flanked by a C-terminal tail and preceded by a highly variable N-terminal region [9, 10]. Several studies have revealed that it is the "α-crystallin domain" in sHSPs that leads to the formation of large oligomeric assembly having oligomeric mass ranging between 200–800 kDa and is highly important for the subunit-subunit interactions [11–13]. sHSPs bind different client proteins under chemical and thermal stress and protect them from misfolding and aggregation. They also help in refolding of stressed proteins and are therefore known as molecular chaperones [14, 15].

Several small heat shock proteins have been reported to have increased chaperone function with increasing temperature [16–18]. This alteration in the chaperone function of small heat shock proteins is attributed to many factors, such as perturbation in oligomeric assembly, availability of hydrophobic patches, increased subunit exchange rate etc. For example, archaeal sHSPs (HSP16.5 from *Methanococcus jannaschii* and HSP26 from *Saccharomyces cerevisiae*), mammalian sHSPs (α-crystallin and HSP27 from human), plant sHSPs (HSP17.2 and HSP17.9 from sugarcane and HSP16.9 from wheat) and mycobacterial sHSPs (HSP16.3 from *Mycobacterium tuberculosis*) are some of the best characterised sHSPs, whose chaperone function was found to increase with increasing temperature [16–22]. Most of them have revealed that it is the dissociation of the oligomeric assembly of sHSPs (HSP26, α-crystallin, HSP17.2, HSP17.9 and HSP16.9) under environmental stress (especially enhanced temperature) that activates the chaperone function of these sHSPs [20, 21, 23]. In contrast, the enhanced chaperone function of HSP27 with increasing temperature has been attributed to the association of its oligomeric assembly [17]. Contradictory to the above examples, some studies have also shown that with increasing temperature for certain sHSPs (HSP16.3 and HSP16.5), though an increase in chaperone function have been observed but its oligomeric size remained unaltered [16, 24]. The increased chaperone activity of such sHSPs with increasing temperature has not been due to any changes in its static oligomeric size but rather due to changes in dynamics of oligomeric assembly such as enhanced rate of subunit exchange [16, 24]. Besides the perturbation in oligomeric assembly caused due to temperature rise, a plethora of studies have also been investigated on the nature of forces that are employed by these sHSPs to bind the aggregation prone substrate proteins under thermal stress. Majority of these studies have shown hydrophobic interactions to be the driving force to bind the aggregation prone substrate proteins, which is a leading cause for the enhanced chaperone activity of sHSPs with increasing temperature [18, 25]. However, HSP16.3 and αB-crystallin are exceptions to such observations and exhibits decreased surface hydrophobicity with increasing temperature [16, 26]. It has been further shown that it is the electrostatic interaction rather than hydrophobic interaction playing a crucial role in the chaperone function of HSP16.3 and αB-crystallin [27, 28]. In summary, it is very much evident from the above instances that the static or dynamic properties of oligomeric assembly and nature of forces, governs the enhanced chaperone activity of sHSPs under thermal stress.

Recently, we have revealed that *Mycobacterium leprae* HSP18 (*M*. *leprae* HSP18), a class 3 heat shock protein, efficiently prevents the aggregation of substrate proteins under thermal and chemical stress [29]. In that same paper, we have shown that *M*. *leprae* HSP18 exists in large oligomeric assembly (~29mer). It is basically an immunodominant antigen [30] which is specifically activated during intracellular growth and is involved in the survival of *M*. *leprae* pathogen within macrophages [31]. It is believed that the molecular chaperone function of HSP18 may play a crucial role in the long term survival of *M*. *leprae* pathogen in infected hosts [32]. But the mechanism behind the chaperone function of HSP18 is still not properly understood. Therefore, to understand the mechanism by which HSP18 prevents the aggregation of different client proteins from aggregation, we investigated the effects of heat on the conformation and chaperone activity of HSP18 using several biophysical and biochemical techniques. We found that heat stress alters the dynamics of oligomeric assembly and enhances the chaperone activity of *M*. *leprae* HSP18. Our results also provide the clue about the mode of interaction between HSP18 and the client proteins at elevated temperature.

## Materials and Methods

### Materials

Bovine insulin, citrate synthase (CS) from porcine heart, Dithiothreitol (DTT) and 4,4'-dianilino-1,1'-binaphthyl-5,5'-disulfonic acid, dipotassium salt (bis-ANS) were purchased from Sigma Chemical Co. (St. Louis, MO, USA). Alexa fluor 350 and Alexa fluor 488 protein labeling kits (Catalog Nos. A-10170 and A-10235, respectively) were obtained from Molecular Probes (Invitrogen, Carlsbad, CA, USA). Malate dehydrogenase (MDH) from porcine heart, disodium hydrogen phosphate and mono sodium hydrogen phosphate were from Sisco Research Laboratories, India. All other chemicals were of analytical grade.

### Expression and purification of recombinant *M*. *leprae* HSP18

pQE31 expression vector harbouring HSP18 gene was obtained as a gift from Prof. Dharmalingham (Madurai Kamraj University, Madurai, India). Methods of expression and purification of wild-type *M*. *leprae* HSP18 has been described previously [29]. Concentration of HSP18 was determined spectrophotometrically by measuring absorbance at 278 nm using extinction coefficient of 0.4 (mg/ml)^-1^cm^-1^. Concentration of this protein was also determined using Bradford assay. Molar concentration of HSP18 was calculated using its monomeric form. All biophysical assays were repeated with three different protein preparations.

### *In vitro* aggregation assay

#### Insulin aggregation assay

The chaperone activity was determined with insulin as client protein (CP) as previously described [29]. Briefly, insulin (0.35 mg/ml) in 50 mM phosphate buffer (pH 7.5) was incubated in the absence and presence of 0.35 mg/ml HSP18. Aggregation of insulin B-chain was initiated by adding DTT at different temperatures (25. 31, 37 and 43°C) and light scattering was recorded at 400 nm at each of these temperatures for 1 hr in the kinetic mode with a data pitch of 10 seconds using Perkin Elmer UV spectrophotometer (Boston, MA, USA) equipped with temperature regulated water bath. The final concentration of DTT in cuvette was 20 mM. We also performed the similar experiments in 50 mM phosphate buffer (pH 7.5) containing 0.05–0.5 M sodium chloride (NaCl). Percent (%) protection for these aggregation assays was calculated using the following equation:

$$\%\text{Protection}=\frac{\left[{\left(O.D._{60\min}\right)}_{CP}-{\left(O.D._{60\min}\right)}_{CP+HSP18}\right]}{{\left(O.D._{60\min}\right)}_{CP}}X100$$ Eq 1

Data are the mean ± standard deviation (SD) from triplicate determinations.

#### Citrate synthase aggregation assay

The chaperone activity of HSP18 in the absence or presence of NaCl was also determined using heat induced aggregation assay of citrate synthase (CS) at 43°C with the aid of same spectrophotometer [33]. Briefly, HSP18 was pre-incubated with 0.05–0.5 M NaCl for 1 hr at 25°C. CS (0.06 mg/ml) aggregation was initiated by heating at 43°C in 50 mM phosphate buffer (pH 7.5) in the absence or presence of HSP18 pre-incubated without or with 0.05–0.5 M NaCl. The CS aggregation was monitored at 400 nm at 43°C for 1 hr in the kinetic mode using a data pitch of 10 seconds. Percent (%) protection for this aggregation assay was calculated using Eq 1. Data are the mean ± SD from triplicate determinations.

### Thermal deactivation assay

Thermal deactivation of malate dehydrogenase (MDH) was performed as previously described [33]. Briefly, deactivation of 10 nM MDH in 50 mM phosphate buffer, pH 7.5 was initiated by heating the enzyme at 43°C in the absence or presence of 30 μM HSP18 pre-incubated with/without 0.05–0.5 M NaCl. MDH enzyme activity was assayed as described previously [29, 33] by collecting aliquots from the assay mixture incubated at 43°C for 10 mins. Experiments were also performed by replacing HSP18 with BSA to check specificity. Data are the mean ± SD from triplicate determinations.

### bis-ANS fluorescence measurements

Fluorescence of bis-ANS was measured as described previously [29] using Fluoromax 4P (Horiba Jobin Mayer, USA) spectrofluorimeter equipped with a thermostated cell holder. Fluorescence spectra of bis-ANS (10 μM) bound to HSP18 (0.05 mg/ml) were taken after incubation for 1 hr at 25, 31, 37 and 43°C. An excitation wavelength of 390 nm was used and the excitation and emission slits were 2.5 nm and 5 nm respectively. Fluorescence emission spectra were recorded in the range of 450–550 nm at the respective temperatures with a data pitch of 0.5 nm. The scan rate used for this assay was 240 nm/min. Each data point is the mean of triplicate measurements.

### Measurements of tryptophan fluorescence

Tryptophan fluorescence spectra of HSP18 (3 μM) in 50 mM phosphate buffer, pH 7.5 were recorded from 310–400 nm at 25, 31, 37 and 43°C with a data pitch of 0.5 nm using spectrofluorometer (Fluoromax 4P, Horiba Jobin Mayer, USA) [29]. The excitation wavelength was set to 295 nm. The scan rate used for this assay was 240 nm/min. Each data point is the mean of triplicate measurements.

### Circular dichorism (CD) measurements

Far- and near-UV CD spectra of HSP18 at different temperatures between 25–43°C were recorded using Chirascan CD spectropolarimeter (Applied Photophysics, Leatherhead, UK) equipped with peltier system as described previously [29]. Protein concentrations used in far- and near-UV CD experiments were 0.2 mg/ml (in 10 mM phosphate buffer, pH 7.5) and 0.5 mg/ml (in 50 mM phosphate buffer, pH 7.5), respectively. Far-UV CD spectra were collected from 195–260 nm using a quartz cell with 1 mm path length, while the near-UV CD spectra were collected from 250–300 nm using 10 mm path length cell. Scan rate of 50 nm/min, data pitch of 0.5 nm, band width of 1 nm and response time of 0.2 seconds were used for far- and near- UV CD data collection. The reported spectra for far- and near-UV CD are the mean of five scans. For both experiments, spectra were recorded after incubating HSP18 at respective temperature for 1 hr. The curve-fitting program CONTINLL was used to analyze the secondary structural content of HSP18 at different temperatures [29].

### Hydrodynamic radius (R_H_) measurements by DLS

*M*. *leprae* HSP18 (0.5 mg/ml, in 50 mM phosphate buffer, pH 7.5) was filtered through 0.22 μm disk membrane. Hydrodynamic radius of HSP18 oligomers pre-incubated without/with 0.5 M NaCl was measured by dynamic light scattering (DLS) employing a Zetasizer Nano S (Malvern Instruments, Malvern, UK) at 25, 31, 37 and 43°C with 1 hr incubation at each temperature prior to reading [34]. Hydrodynamic radius of HSP18 oligomers was also measured at 25°C after pre-incubating the protein with 0.05, 0.15 and 0.5 M NaCl for 1 hr. During measurement, He–Ne laser (4 mW; 633 nm) was used and the detector angle was fixed at 173°. Every run composed of 12 acquisitions. Each data is an average of 5 such runs. R_H_ was calculated of using the Stokes–Einstein equation:

$$R_{H}=\frac{k_{B}T}{6\pi \eta D}$$ Eq 2

where, k_B_ is Boltzmann’s coefficient, T is absolute temperature, η is the viscosity of the medium and D is translational diffusion coefficient of the particles [35].

### Estimation of oligomeric mass using gel filtration chromatography

Oligomeric mass of HSP18 in the absence or presence of different NaCl concentrations (0.05, 0.15 and 0.5 M) was determined at 25°C using gel filtration chromatography as described previously [29]. All these estimations were done in HPLC (Dionex, Sunnyvale, CA, USA) using an analytical gel filtration column (TSK-GEL G4000SW_XL_). Protein concentration used was 0.5 mg/ml in 50 mM phosphate buffer, pH 7.5. Calibration of the column was performed using different molecular weight standards: thyroglobulin (669 kDa), apoferritin (443 kDa), β-amylase (200 kDa), BSA (66 kDa) and carbonic anhydrase (29 kDa) (Sigma Chemical Co.). Flow rate used for all measurements were 0.5 ml/min.

### Structural stability measurements

The structural stability of HSP18 was determined by both far-UV CD spectroscopy and differential scanning calorimetry (DSC) measurements.

#### Using far-UV CD spectroscopy

The structural stability of HSP18 in the absence or presence of 0.5 M NaCl was determined using thermal denaturation experiments as described previously [29]. Briefly, HSP18 (0.2 mg/ml in 10 mM phosphate buffer, pH 7.5) was heated from 25–80°C with a ramp rate of 0.5°C/min and the alteration in ellipticity at 222 nm was recorded using Chirascan CD spectropolarimeter (Applied Photophysics, Leatherhead, UK). Mid-point transition or melting temperature (T_m_) was estimated using sigmoidal analysis as described previously [29] and van’t Hoff equation was used to calculate the enthalpy change (ΔH_vH_) associated with the thermal transition of HSP18 in the absence and presence of 0.5 M NaCl.

#### Differential scanning calorimetry (DSC)

The excess heat capacity was measured as a function of temperature in a VP DSC Microcalorimeter (GE Healthcare) to investigate thermal denaturation experiments of HSP18 in the absence or presence of NaCl. The samples were scanned from 20°C to 80°C with a scan speed of 60°C/hr at approximately 25 psi pressure. Prior to sample scan, the instrument was thermally stabilized by repeated buffer scans under similar conditions. Protein concentration used was 1 mg/ml in 50 mM phosphate buffer, pH 7.5 to obtain the melting profile of HSP18 in the absence or presence of NaCl. The thermograms obtained were analyzed using the in-built VPViewer software with Origin 7.0. The 2-state model of curve fitting was used to fit the raw thermograms.

### Fluorescence labeling of recombinant *M*. *leprae* HSP18 with Alexa fluor 488 and Alexa fluor 350

Fluorescence conjugation of Alexa fluor 350 and Alexa fluor 488 fluorescent probes with purified recombinant *M*. *leprae* HSP18 was done as described previously [36, 37]. During tagging, we followed the procedure described by the manufacturer (Molecular Probes, Invitrogen, Carlsbad, CA, USA). Briefly, protein solutions (1 mg/ml) supplemented with 100 mM sodium bicarbonate (pH 7.2) were mixed with Alexa fluor probes for 1 hr at 25°C. BioGel P-30 column was used to separate the labeled protein from the unlabeled protein. The fraction, which contained the labeled HSP18, was collected and dialyzed against 50 mM phosphate buffer (pH 7.5) for 24 hr. The amount of HSP18 labeled with Alexa fluor 488 was estimated by measuring the absorbance at 278 and 494 nm of HSP18 and Alexa fluor 488, respectively. The degree of fluorescence labeling of Alexa fluor 350 to HSP18 was determined from the absorbance of HSP18 and Alexa fluor 350 at 278 and 346 nm, respectively. Concentrations of Alexa fluor 488 and Alexa fluor 350 were determined spectrophotometrically with the aid of molar extinction coefficients of 71,000 cm^-1^M^-1^ at 494 nm and 19,000 cm^-1^M^-1^ at 346 nm, respectively.

### Measurements of the subunit exchange rate

Fluorescence resonance energy transfer (FRET) technique was used to determine the rate of subunit exchange as described previously [36, 37]. For, subunit exchange experiments, Alexa fluor 350-labeled HSP18 acts as energy donor and Alexa fluor 488-labeled HSP18 acts as the energy acceptor. The proteins were incubated for 3 hr at different temperatures between 25–43°C in 50 mM phosphate buffer (pH 7.5) in a stoichiometric ratio of 1:1 (M/M). The reaction mixture was excited at 346 nm and the fluorescence emission spectra were taken from 400–600 nm at respective temperature using a Fluoromax-4P spectrofluorometer (Horiba Jobin Mayer, USA). The intensities at 440 nm and 513 nm were measured separately, which were used to calculate the subunit exchange rate constant (*k*) from the following equation:

$$F_{t}/F_{o}=A+Be^{-kt}$$ Eq 3

where *F*_t_ is the fluorescence intensity at 440 nm or 513 nm at different time intervals, *F*_o_ is the fluorescence intensity at 440 nm or 513 nm at *t* = 0, and *k* is the subunit exchange rate constant. Constants *A* and *B* were determined using conditions where *A* + *B* = 1 at *t* = 0 and *A* is the fluorescence intensity at *t* = ∞. Origin 8.0 software (OriginLab, Northampton, MA) was used to determine the subunit exchange rate constant of HSP18 subunits at various temperatures. We also performed the similar experiments in 50 mM phosphate buffer (pH 7.5) containing 0.05–0.5 M NaCl at 37°C.

### Statistical analysis

All values are representative of means ± standard deviation of the number of experiments indicated in the respective figure legends. Student’s *t*-test was used for the analysis of the statistical differences between the groups. Only those data were considered statistically significant, whose *p-values* were found to be ≤ 0.05.

## Results and Discussion

*M*. *leprae* encounters various stresses such as hypoxia, nutrient depletion and oxidative stress during its survival in granulomata and macrophages of host species [38, 39]. Very little information exists in the literature regarding the factors which facilitate the virulence and survival of this pathogen under these stresses. Several antigenic proteins play critical role in the survival of *M*. *leprae* pathogen [40–42]. Some of these antigenic proteins having molecular weight 10–70 kDa, belong to heat shock protein family [43]. Among these antigens, only 18 kDa antigen has been identified as small heat shock protein and termed as HSP18. Cole *et al*. reported the comparative analysis of *M*. *leprae* genome sequence with that of *M*. *tuberculosis* and revealed that, despite a massive gene decay in *M*. *leprae*, HSP18 gene has been retained [44], hinting over the fact that this small heat shock protein may be critical for virulence and survival of this pathogen. As *M*. *leprae* is non-culturable, the role of HSP18 in the survival of surrogate hosts (*E*. *coli* and *M*. *smegmatis*) carrying gene of this protein has been recently investigated under such stressed conditions where it has been found that the expression level of HSP18 increases [45]. It has been also shown that HSP18 facilitated the multiplication of *M*. *smegmatis* within macrophages under conditions of nutrient depletion, hypoxia and oxidative stress [45]. The expression level of HSP18 in recombinant *E*. *coli* has also increased moderately under heat stress [45]. Such over-expression of HSP18 provides thermo-tolerance to *E*. *coli* which has been attributed to its chaperone function. However, the effect of heat on the chaperone function of HSP18 is still unclear. In view of the fact that, the structure and chaperone function of well known sHSPs are affected upon heating [16–20], we hypothesized that heat could alter the structure as well as chaperone function of HSP18.

**Fig 1:**
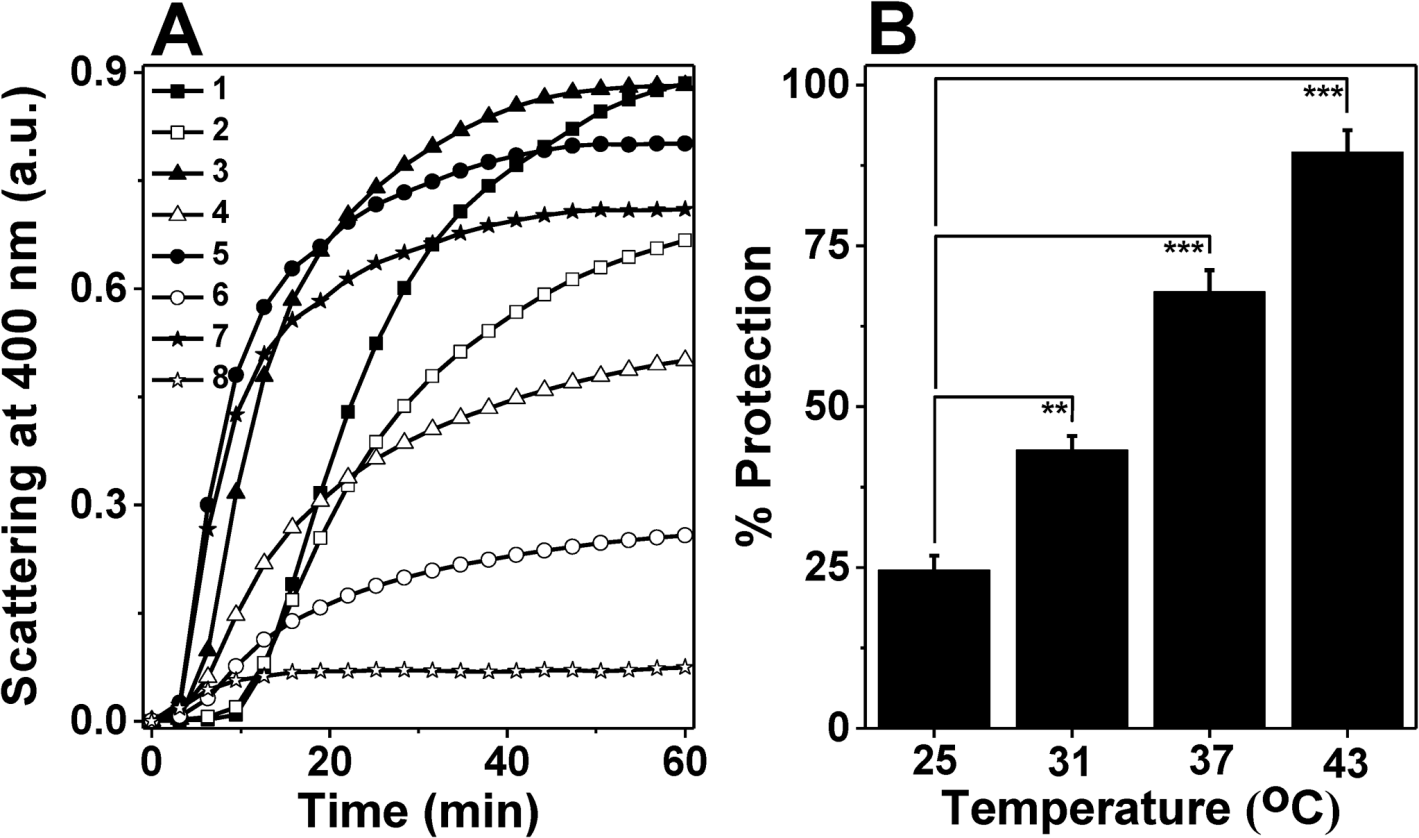
Temperature-dependent chaperone-like activities of *M*. *leprae* HSP18. **(A)** DTT-induced aggregation of 0.35 mg/ml insulin (Ins) in the absence and presence 0.35 mg/ml HSP18 at different temperatures (25, 31, 37 and 43°C). Aggregation was initiated by adding 20 mM DTT and scattering at 400 nm was monitored at the respective temperatures. Trace 1: Ins alone at 25°C; Trace 2: Ins + HSP18 at 25°C; Trace 3: Ins alone at 31°C; Trace 4: Ins + HSP18 at 31°C; Trace 5: Ins alone at 37°C; Trace 6: Ins + HSP18 at 37°C; Trace 7: Ins alone at 43°C; Trace 8: Ins + HSP18 at 43°C; **(B)** Percent protection ability of *M*. *leprae* HSP18 against insulin aggregation at different temperatures. Data are means ± the standard deviation from triplicate determinations. **p< 0.005 and ***p< 0.0005.

To accomplish this, we first investigated the chaperone activity of *M*. *leprae* HSP18 at different temperatures. As the normal body temperature of human being, the host of pathogen *M*. *leprae* is ~37°C, the temperature range that has been selected for this study is 37 ± 6°C i.e. 31, 37 and 43°C. Apart from these three temperatures, room temperature (25°C) was also selected. Effect of heat/temperature on the chaperone function of HSP18 was assessed by monitoring the ability of HSP18 to prevent aggregation of insulin B chain. It was co-incubated with insulin at different temperatures (25, 31, 37 and 43°C) for 1 hr and DTT induced insulin aggregation assay was performed at the respective temperatures. At a ratio of 1:1 (w/w) of HSP18 to insulin, 24.6% protection against insulin aggregation was observed at 25°C (Fig 1A, trace 2 and 1B). Surprisingly, at the same chaperone to client protein ratio, the protection was increased to ~43% at 31°C (Fig 1A, trace 4 and 1B). When the temperature was further increased to 37°C, a further increase in the protection against insulin aggregation was observed (~68%) (Fig 1A, trace 6 and 1B). HSP18 protected substantially/almost the insulin aggregation (90%) at 43°C (Fig 1A, trace 8 and 1B). Therefore, it is quite evident from here that the temperature has profound influence on the aggregation prevention ability of HSP18. The chaperone function of HSP18 increases with increasing temperature.

**Fig 2:**
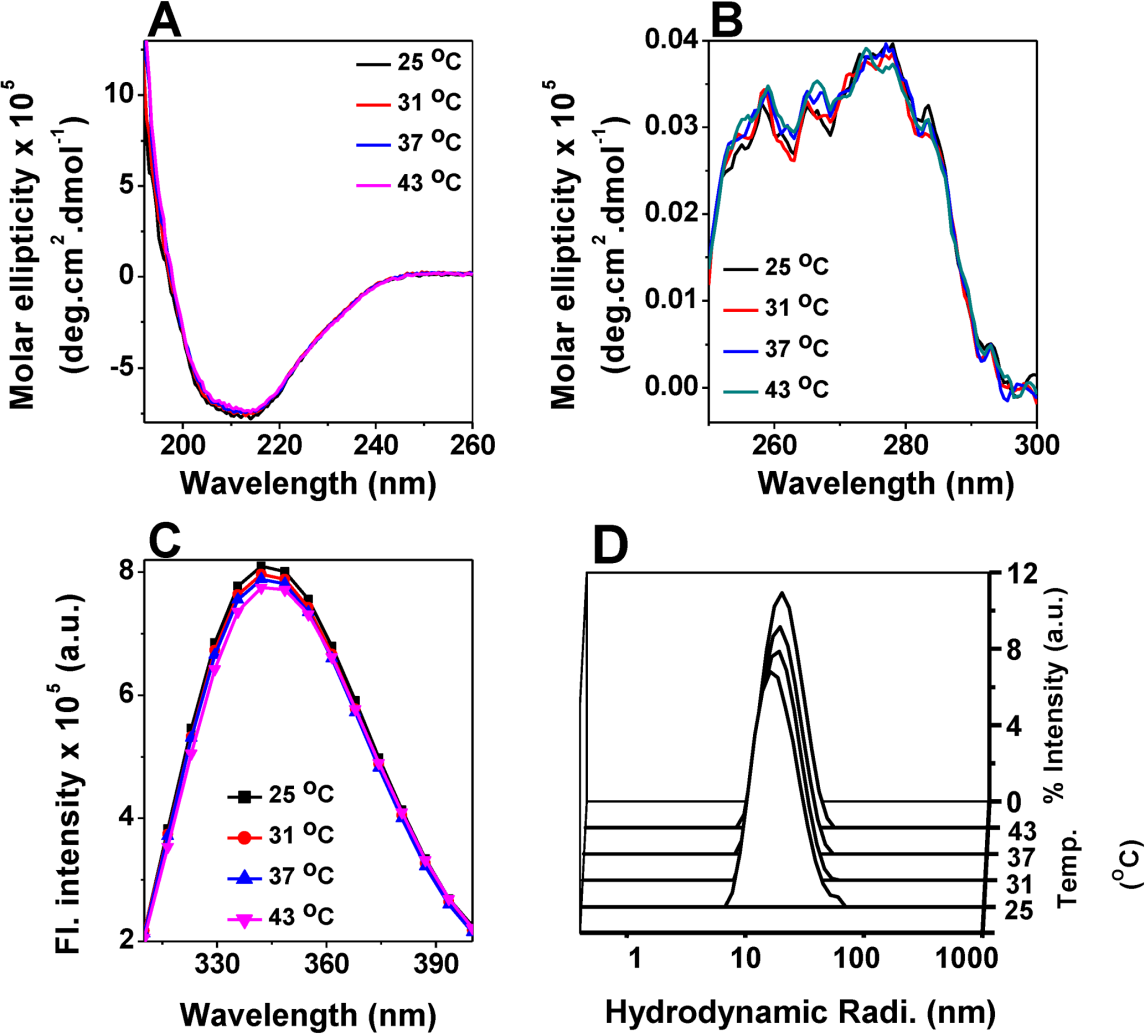
Effect of temperature on the structure of *M*. *leprae* HSP18. Far-UV CD spectra **(A)** and near-UV spectra **(B)** of HSP18 at different temperatures (25, 31, 37 and 43°C). The concentrations of the protein samples used in far- and near-UV CD experiments were 0.2 and 0.5 mg/ml, respectively. **(C)** Tryptophan fluorescence spectra of HSP18 (0.05 mg/ml) were recorded from 310–400 nm at various temperatures (25, 31, 37 and 43°C). An excitation wavelength of 295 nm was used. Both the slit widths for excitation and emission were 5 nm. Data were collected at 0.5 nm wavelength resolution. **(D)** Intensity particle size distribution spectra of *M*. *leprae* HSP18 were recorded at various temperatures (25, 31, 37 and 43°C). Each of these spectra is an average of 48 scans. For each experiment, spectra were recorded after incubating HSP18 at respective temperature for 1 hr.

In order to understand whether the enhancement in the chaperone activity of HSP18 at elevated temperature is accompanied by any alteration in the proteins’ conformations, secondary, tertiary and quaternary structure/conformation was studied at different temperatures (25, 31, 37 and 43°C). Far-UV CD spectra of HSP18 at these four temperatures are shown in Fig 2A, which clearly indicate that temperature has no effect on the secondary structure of HSP18. Data represented in Table 1 clearly indicated that HSP18 is a major β-sheet protein at 25°C. However, with increasing temperature, no change in the secondary structural elements of HSP18 was observed (Table 1). These data further confirmed that secondary structure of HSP18 was not altered upon the temperature rise from 25°C to 43°C. In addition, the effect of temperature/heat on the tertiary structure of HSP18 was studied using near-UV CD experiment (Fig 2B). The signal between 250–270 nm region arose from the five phenylalanine residues in HSP18 (Fig 2B). The signal beyond 270 nm corresponds to the single tyrosine and tryptophan residue in HSP18 (Fig 2B). When the near-UV CD spectra of HSP18 was recorded at elevated temperatures i.e. 31, 37 and 43°C, the spectral characteristics remained unaltered compared to that recorded at 25°C (Fig 2B). These data indicated that microenvironment of aromatic amino acid residues in HSP18 was not perturbed due to increase in temperature. The intrinsic tryptophan fluorescence spectra of HSP18 at different temperatures (25, 31, 37 and 43°C) agreed with the near-UV CD results (Fig 2C). No alteration in λ_max_ as well as intensity of the intrinsic tryptophan fluorescence spectra was observed at higher temperature which further confirmed that temperature did not perturb the microenvironment of the single tryptophan residue (W33) of HSP18.

**Table 1:** Secondary structural elements of HSP18 at various temperatures.

Temperature (°C)	α-Helix (%)	β-Sheet (%)	β-Turn (%)	Random Coil (%)
25	4.1	42.5	21.6	31.8
31	4.2	42.4	21.5	31.9
37	4.2	42.6	21.4	31.8
43	4.2	42.5	21.5	31.8

Oligomeric structure of sHSPs is known to play a key role in its chaperone function. Several reports have revealed that increase in temperature have altered the oligomeric size of sHSPs [17, 21, 23]. Thus the oligomeric state of HSP18 was studied using dynamic light scattering (DLS) experiment between 25°C to 43°C. Fig 2D shows the intensity particle size distribution spectra of HSP18 at temperatures between 25–43°C. It was observed that at 25°C, the size distribution spectra of HSP18 was centred around 18.2 nm which corresponded to a molecular weight of 588 kDa. When HSP18 was incubated at higher temperatures (31, 37 and 43°C), no change in hydrodynamic radius was observed (Fig 2D). HSP18 could hold its native oligomeric size even at 43°C. This led us to conclude that temperature/heat did not perturb the oligomeric/quaternary structure of *M*. *leprae* HSP18. Overall, these findings revealed that the secondary, tertiary and quaternary structure of the protein remains unaltered in this temperature range.

**Fig 3:**
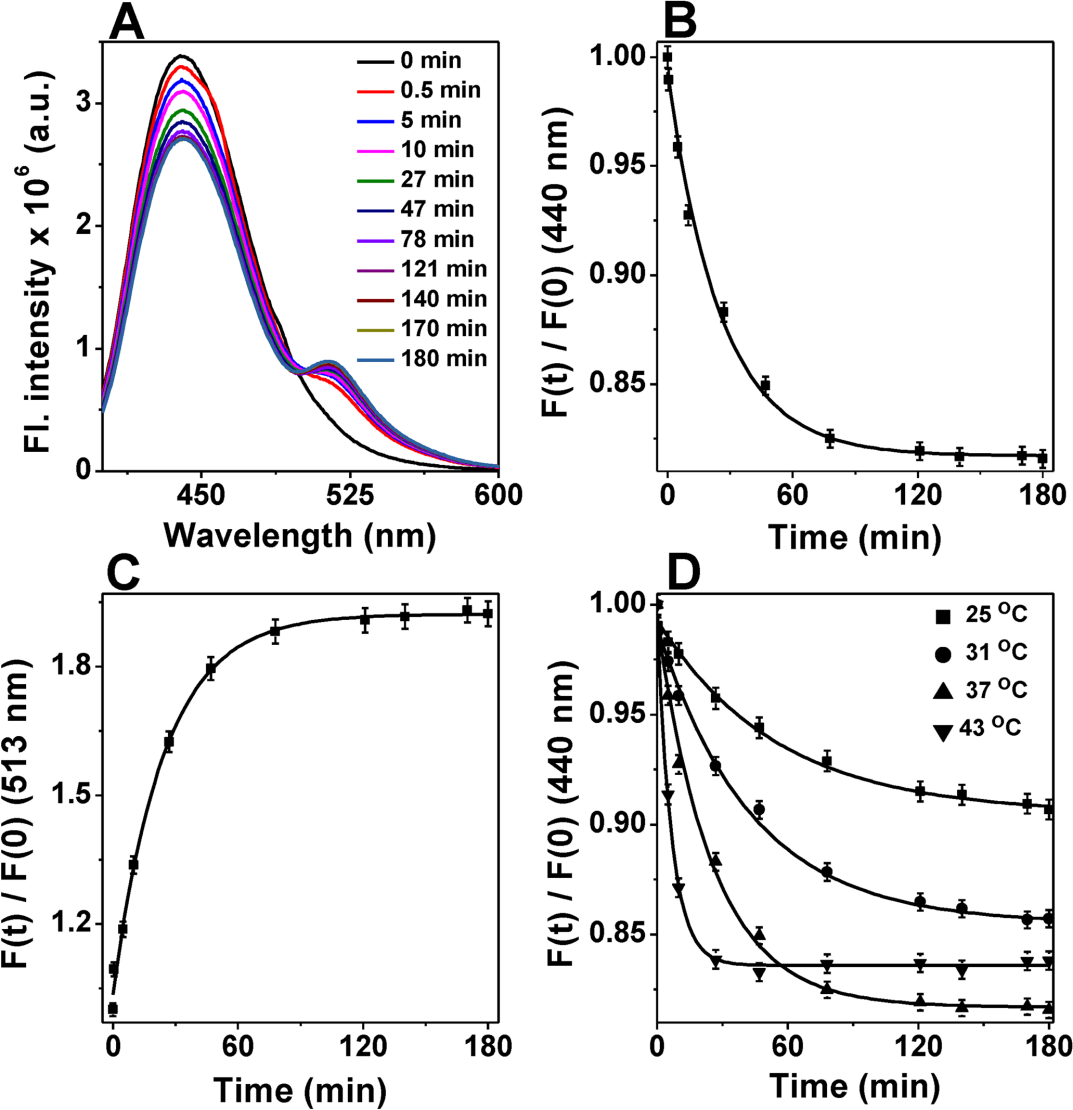
Determination of subunit exchange rate constant of HSP18 subunits at different temperatures. **(A)** Time-course alterations in the emission spectrum of Alexa fluor-350 and 488 labeled *M*. *leprae* HSP18 due to subunit exchange at 37°C. The emission spectra were recorded at different time points after mixing equal amount of Alexa fluor-350 labeled and Alexa fluor-488 labeled HSP18 (1 mg/ml each) at 37°C. The fluorescence spectra were recorded from 400 to 600 nm at 37°C using the excitation wavelength of 346 nm. The slit width of both excitation and emission monochromators was 5 nm each. The scan rate used for this assay was 240 nm/min. **(B)** Time-dependent decrease in fluorescence intensity at 440 nm for data shown in panel A. **(C)** Time-dependent increase in fluorescence intensity at 513 nm for data shown in panel A. **(D)** Temperature-dependent subunit exchange rate of *M*. *leprae* HSP18. Subunit exchange between Alexa fluor-350 labeled and Alexa fluor-488 labeled HSP18 was monitored at 25, 31, 37 and 43°C. Symbols in panel B, C and D represent the experimental data points and the solid lines in these pannels represent the best fit of the data according to Eq 3.

**Fig 4:**
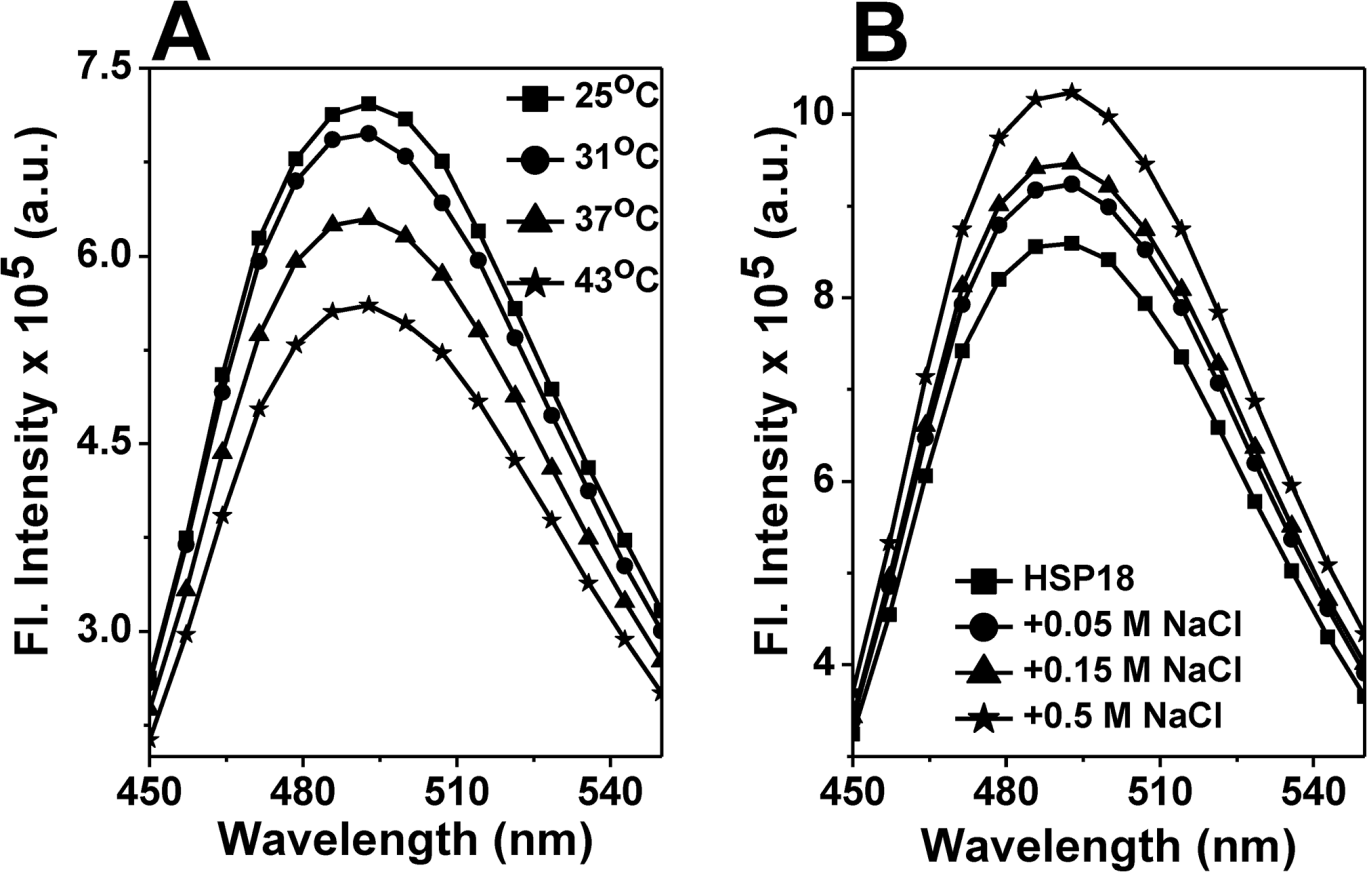
bis-ANS binding to *M*. *leprae* HSP18. **(A)** Fluorescence spectra of bis-ANS bound to HSP18 at different temperatures; **(B)** Fluorescence spectra of bis-ANS bound to HSP18 in the absence or presence of various concentrations of NaCl at 25°C. Protein concentration in all samples was 0.05 mg/ml and bis-ANS concentration was 10 M. The excitation wavelength was 390 nm. Excitation and emission slit widths were 2.5 and 5 nm, respectively. All the reported spectra were obtained by subtracting the respective control (bis-ANS alone) curves/spectra from the spectra of bis-ANS bound to HSP18.

The hallmark of sHSPs is to assemble into large oligomeric assembly of high molecular mass. Often subunits of different sHSPs exchange with each other by dynamic oligomeric dissociation/re-association which is pre-requisite for their chaperone function [16, 24, 37]. Since static oligomeric structure of HSP18 remained unaltered at elevated temperatures (31–43°C), we hypothesised that it is the changes in dynamics of oligomeric structure that caused enhanced chaperone function of HSP18 at these higher temperatures. First, we labelled HSP18 separately with Alexa fluor 350 (A-350) and Alexa flour 488 (A-488) and determined the degree of labeling. We observed that percentage labelling of Alexa fluor 350 and Alexa fluor 488 to HSP18 was 1:1 (M/M) which suggests that all proteins were labelled with these fluorophores. Size exclusion chromatographic experiments revealed that these labelling did not perturb the static oligomeric assembly and exhibited similar oligomeric mass/size compared to that of unlabelled HSP18 (S1 Fig). Then, we monitored the subunit exchange reaction at physiological temperature (37°C) by mixing an equimolar concentration of the donor, Alexa flour 350-labelled HSP18, with the acceptor, Alexa flour 488-labelled HSP18. Due to exchange between subunits of HSP18, we observed marked alteration in the donor and acceptor fluorescence (Fig 3A). Because of FRET, a time dependent decrease in fluorescence intensity at 440 nm for A-350 (labelled to HSP18) was observed with a concomitant time dependent increase in fluorescence intensity at 513 nm for A-488 (labelled to HSP18) which reached to its saturation in 3 hr at 37°C (Fig 4). To find out the rate constant (k) for the subunit exchange, the declining fluorescence intensity of A-350 (Fig 3B) and increasing fluorescence intensity of A-488 (Fig 3C) was fitted to the Eq 3. In both cases, we found that the rate constant (k) for the subunit exchange at 37°C was 0.039 min^-1^. This value of subunit exchange rate constant for HSP18 is almost comparable with the value of 0.0378 min^-1^ reported by Bova *et al*. [46] for the subunit exchange of αA-crystallin at 37°C. In the same paper, they also determined the rate constant for the subunit exchange of HSP27 at 37°C (0.0576 min^-1^) which is slightly higher compared to that of HSP18 and αA-crystallin. To understand the effect of temperature on the subunit exchange rate of HSP18, rate constant (k) of subunit exchange was also determined at 25, 31 and 43°C (Fig 3D). The subunit exchange rate constants for HSP18 at these three temperatures were 0.018, 0.023 and 0.149 min^-1^, respectively (Table 2 and Fig 3D). Thus, increasing the temperature from 25°C to 43°C, the magnitude of subunit exchange rate constant for HSP18 was increased almost 8 fold. Moreover, the subunit exchange rate constant was found to markedly increase (~4 fold) for HSP18 when the temperature was raised from 37°C to 43°C. Interestingly, almost similar rise in temperature (37°C to 42°C) also increased the magnitude of subunit exchange rate constant for αA-crystallin by ~4.2 fold [47]. Moreover, we can say that HSP18 possess a dynamic quaternary structure even at 25°C and dynamics of oligomeric dissociation/re-association becomes rapid at elevated temperatures. In several previous studies, investigators demonstrated that the enhancement of the chaperone function of various sHSPs under elevated temperature is mostly associated with increased subunit exchange [16, 24, 47]. Therefore, we concluded that increased subunit exchange of HSP18 subunits at elevated temperature may be one of the basis for the enhancement of its chaperone function at higher temperature.

**Table 2:** Subunit exchange rate constant of *M*. *leprae* HSP18 at different temperatures.

Temperature at which subunit exchange kinetics/reaction was done (°C)	Subunit exchange rate constant (k) (min^-1^)
25	0.018 ± 0.002
31	0.023 ± 0.003
37	0.039 ± 0.005
43	0.149 ± 0.004

Apart from change in the kinetics of subunit exchange reaction, the alteration in the surface hydrophobicity of HSP18 at different temperatures was also determined. Generally, sHSPs possess several hydrophobic patches at the surface and these patches recognise and bind aggregation prone substrate proteins through hydrophobic interactions. It is usually believed that surface hydrophobicity governs the chaperone function of different sHSPs [48–51]. Additionally, several reports in the literature showed that the enhancement in the chaperone function of sHSPs is often accompanied by increased surface hydrophobicity [48, 49, 51]. The surface hydrophobicity of HSP18 at different temperatures (25, 31, 37 and 43°C) was estimated using bis-ANS. This hydrophobic probe is widely used for probing the hydrophobic sites of several sHSPs [48, 49, 51]. We observed that the fluorescence intensity of bis-ANS bound to HSP18 decreased marginally (~5%) during the rise in temperature from 25°C to 31°C (Fig 4A). We also noticed that the fluorescence intensity of bis-ANS bound to HSP18 exhibited gradual decrease (12% and 22%) upon heating at 37°C and 43°C, respectively (Fig 4A). All these data indicated that the availability of hydrophobic patches at the surface of HSP18 is reduced at elevated temperature. Thus, it seems that HSP18 is more hydrophobic at lower temperature and less hydrophobic at higher temperature. Also, we can conclude that hydrophobic interaction may not be the determinant factor behind the enhanced chaperone activity of HSP18 at higher temperature.

**Fig 5:**
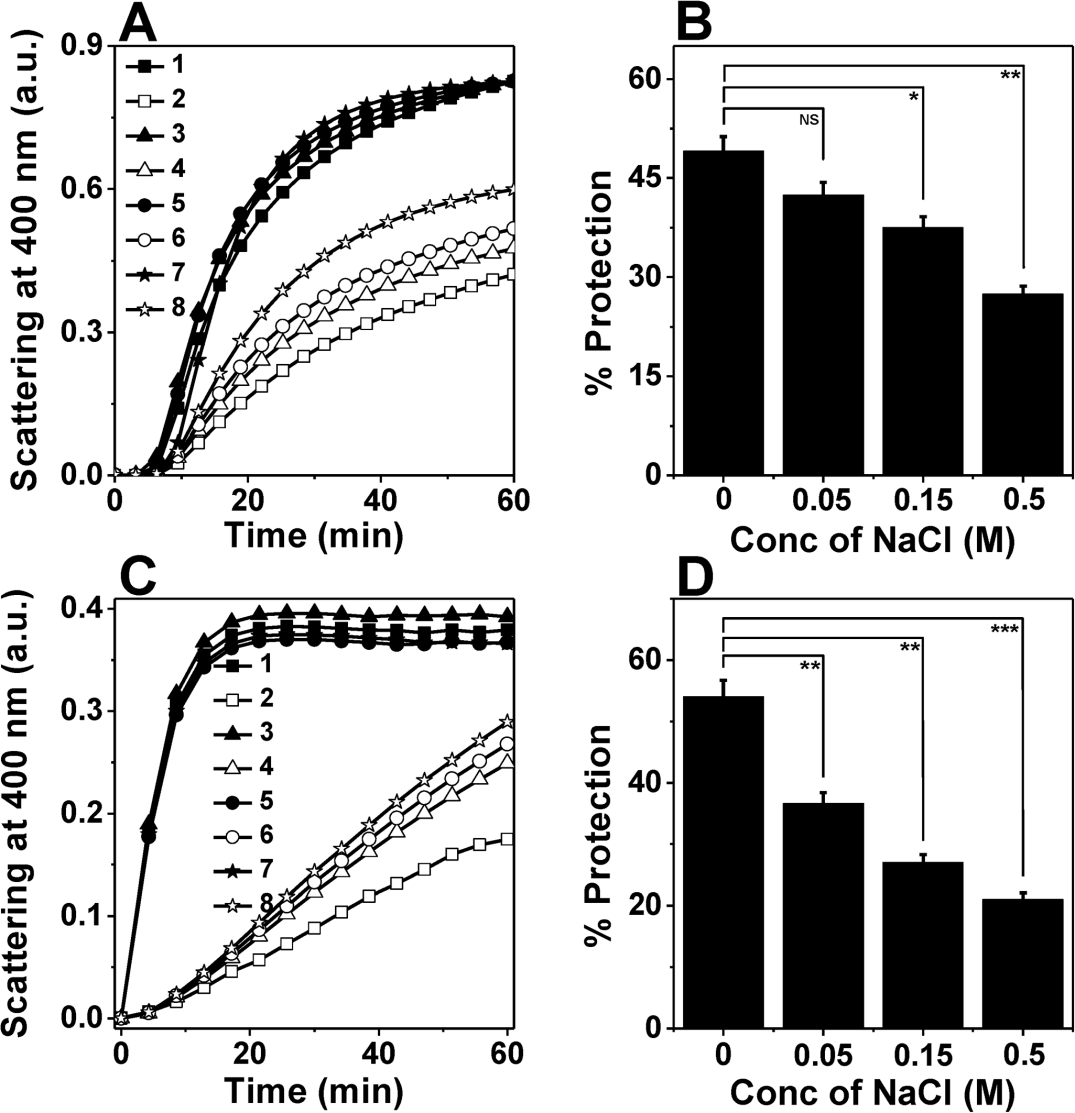
Effect of NaCl on the chaperone activity of *M*. *leprae* HSP18. DTT-induced aggregation of 0.35 mg/ml insulin at 25°C **(panel A)** and thermal aggregation of 0.06 mg/ml CS at 43°C **(panel C)** in the absence or presence of different HSP18 samples. Both insulin and citrate synthase are denoted as client proteins. Trace 1: Client protein (CP) alone; Trace 2: CP +HSP18; Trace 3: CP +0.05 M NaCl; Trace 4: CP + HSP18 + 0.05 M NaCl; Trace 5: CP +0.15 M NaCl; Trace 6: CP + HSP18+0.15 M NaCl; Trace 7: CP +0.5 M NaCl; Trace 8: CP + HSP18+0.5 M NaCl. Each data point is the average of triplicate measurements. The percent protection ability of different HSP18 samples against insulin and CS aggregation are presented in panels B and D, respectively. The insulin: HSP18 ratio was 1:1.2 (w/w) and the CS: HSP18 ratio was 1:1.5 (w/w). Data are means ± standard deviation from triplicate determinations. NS = Not significant, *p< 0.05, **p< 0.005 and ***p< 0.0005.

**Fig 6:**
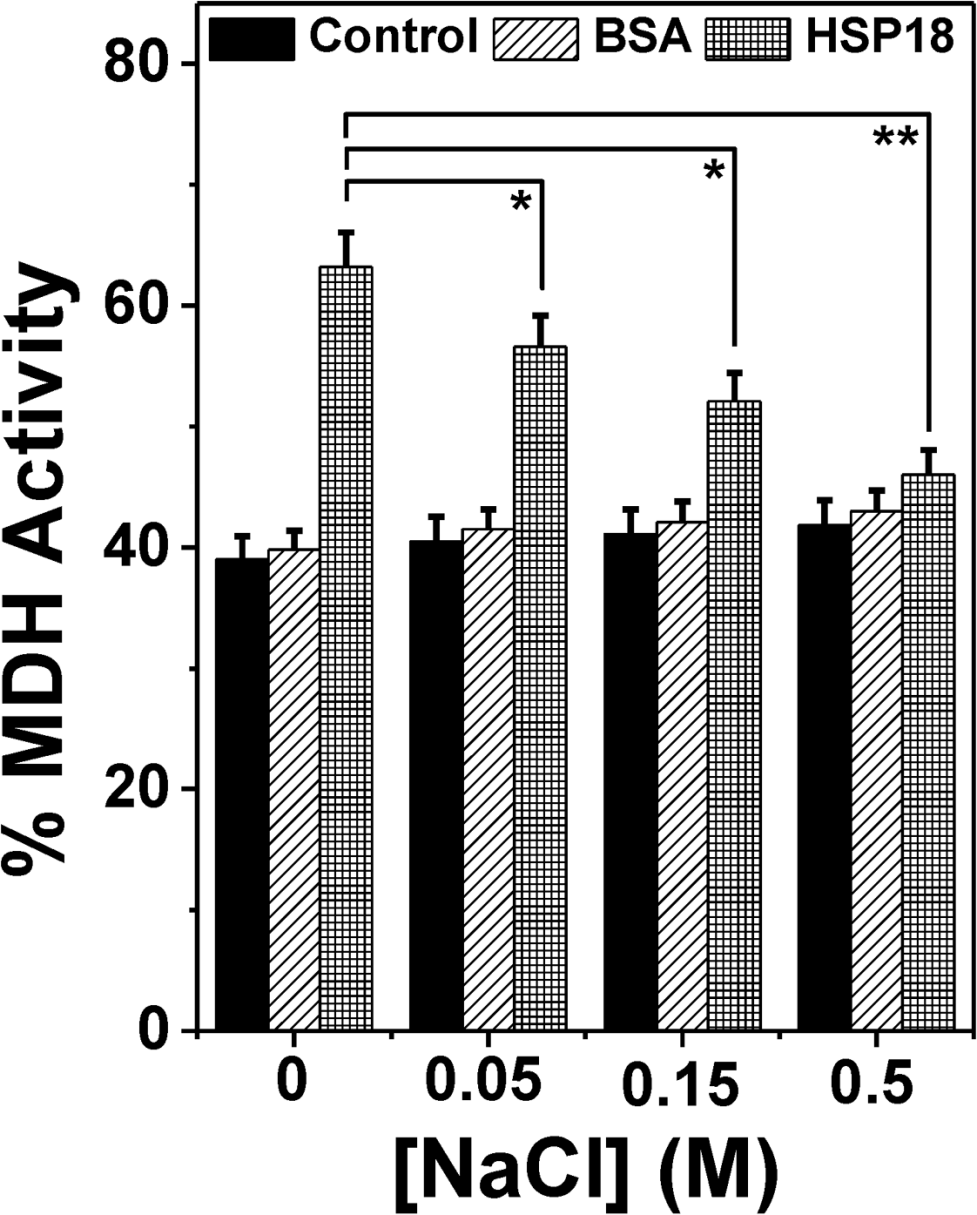
Effect of NaCl on the thermal deactivation prevention ability of *M*. *leprae* HSP18. The MDH enzyme activity was measured in the absence and presence of 30 μM HSP18 pre-incubated in the absence or presence of 0.05–0.5 M NaCl while it was thermally denatured at 43°C. Similar assays were also performed with 30 μM BSA alone or pre-incubated with 0.05–0.5 M NaCl. Data are means ± standard deviation from triplicate determinations. *p< 0.05, **p< 0.005.

Failing to find a direct correlation between chaperone function and surface hydrophobicity in this temperature range prompted us to investigate some other forces which may be responsible for the enhanced chaperone function of HSP18 at higher temperature. In some of the sHSPs (α-crystallin and *M*. *tuberculosis* HSP16.3) it has been reported that, besides the hydrophobic interactions, the chaperone function of these sHSPs are also dependent on the electrostatic/ionic interactions [27, 28]. Charge-charge/electrostatic interactions are key to the substrate binding properties of these sHSPs. Therefore, to understand whether electrostatic interactions play a role in the chaperone function of *M*. *leprae* HSP18, we investigated the chaperone activity of HSP18 in the absence or presence of NaCl (0.05–0.5M) using DTT induced aggregation of insulin at 25°C. The effect of different concentrations of NaCl on the aggregation of insulin in the absence or presence of HSP18 is shown in Fig 5A. At 1:1.2 (w/w) ratio of insulin to HSP18, 49% protection against aggregation was observed in the absence of NaCl (Fig 5A, trace 2 and 5B). In the presence of 0.05 M NaCl, protection was reduced to ~42% (Fig 5A, trace 4 and 5B). As the NaCl concentration was increased from 0.05 M to 0.5 M, the protection ability of HSP18 was found to decrease further (~27%) (Fig 5A, trace 8 and 5B). The insulin aggregation assay was also carried out at 25°C by varying the ratio between HSP18 and insulin and 0.15 M NaCl. In both cases, the chaperone activity of HSP18 was found to decrease in the presence of 0.15 M NaCl (S2 Fig). To check, whether the effect is substrate specific or not, we examined the aggregation prevention ability of HSP18 in the absence and presence of NaCl (0.05–0.5 M) using another client protein i.e. citrate synthase (CS). Usually, CS tends to aggregate upon heating at 43°C. At 1:1.5 (w/w) ratio of CS to HSP18, 54% protection was observed in the absence of NaCl (Fig 5C, trace 2 and 5D). However, in the presence of 0.05 M NaCl, the protection ability was reduced to ~36% (Fig 5C, trace 4 and 5D). Upon further increase in NaCl concentrations (0.15 and 0.5 M), the protection ability was further reduced (27% and 21%, respectively) (Fig 5C, traces 6 & 8 and 5D). Thus from both the aggregation assays it was inferred that HSP18 exhibits reduced chaperone activity in the presence of NaCl. Besides aggregation prevention ability, HSP18 can also prevent the thermal inactivation of enzyme [malate dehydrogenase (MDH)] [33]. To understand whether the weakening of electrostatic interactions impair this ability of HSP18, the thermal deactivation of MDH was carried out without or with different HSP18 protein samples (pre-incubated without or with 0.05–0.5 M NaCl concentrations) (Fig 6). MDH alone could retain only 39% of its activity. However, in the presence of 30 μM HSP18, it could retain ~63% enzyme activity (Fig 6). When HSP18 was pre-incubated with 0.05–0.5 M NaCl and then subjected to MDH thermal deactivation assay, the loss of MDH activity was increased and it could only retain ~57% (at 0.05 M NaCl) to ~46% (at 0.5 M NaCl) enzyme activity (Fig 6). So as to check the specificity, parallel experiments were also carried out where HSP18 was replaced with BSA (Fig 6), but, BSA failed to exhibit thermal deactivation prevention ability both in the absence or presence of 0.05–0.5 M NaCl (Fig 6). Appropriate controls were also done to check the enzymatic activity of MDH in the presence of various concentrations of NaCl without HSP18. Thus it was inferred that the thermal deactivation prevention ability of HSP18 was reduced in the presence of NaCl. Besides, the effect of NaCl on the surface hydrophobicity of HSP18 was also monitored using bis-ANS experiment. Interestingly, with varying concentrations of NaCl ranging from 0–0.5 M, the fluorescence intensity of bis-ANS bound to HSP18 was found to increase by ~19% at 25°C (Fig 4B), suggesting over the fact that surface hydrophobicity of HSP18 was enhanced in the presence of NaCl. However, the chaperone function of HSP18 was reduced in the presence of NaCl at the same temperature. Therefore, it can be concluded from these evidences that electrostatic interactions play a crucial role for proper execution of chaperone function by *M*. *leprae* HSP18 at 25°C.

**Fig 7:**
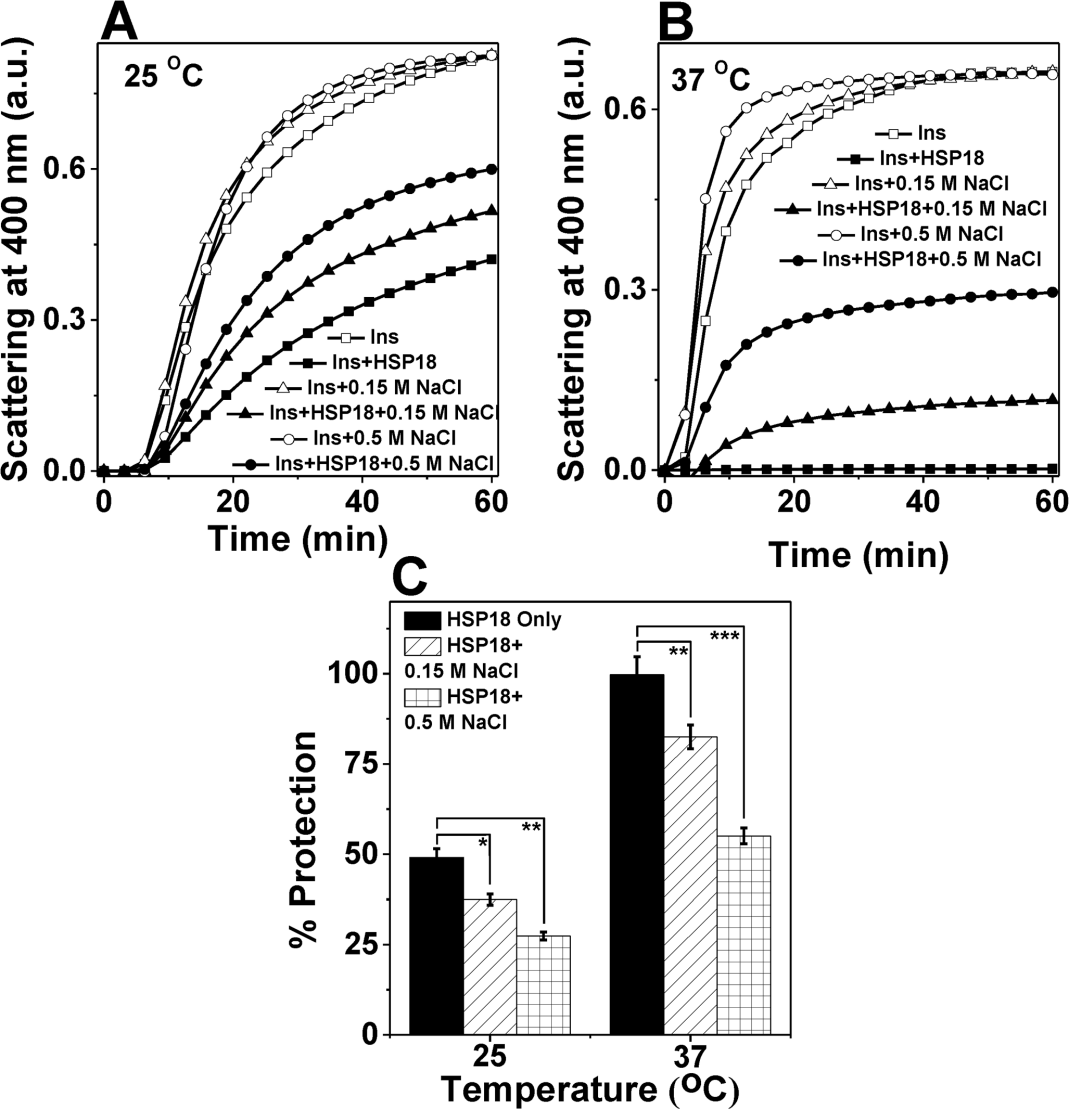
Effect of temperature on the chaperone activity of *M*. *leprae* HSP18 in the presence of NaCl. The aggregation of insulin B chains (0.35 mg/ml) initiated by the addition of DTT (20 mM) was used to determine the chaperone activity of HSP18 (0.42 mg/ml) at **(A)** 25°C and **(B)** 37°C. **(C)** Percentage protection of insulin (0.35 mg/ml) aggregation by HSP18 in the absence or presence of various NaCl concentrations at two different temperatures. Data are means ± the standard deviation from triplicate determinations. *p< 0.05, **p< 0.005 and ***p< 0.0005.

We also checked the chaperone activity of HSP18 at physiological temperature (37°C) in the absence and presence of different NaCl concentrations (0.15 and 0.5 M) using DTT induced insulin aggregation assay. In the presence of 0.15 M NaCl, the chaperone function of HSP18 was found to decrease by ~17.5% at 37°C (Fig 7B and 7C), while the same was found to decrease by ~11.6% at 25°C (Fig 7A and 7C). The more dramatic effect was observed in the presence 0.5 M NaCl. At this NaCl concentration, we noted that the chaperone function of HSP18 was lowered by ~21.6% at 25°C (Fig 7A and 7C). However, at the same salt concentration, the protection ability of HSP18 was reduced significantly (~45%) at 37°C (Fig 7B and 7C). We also found the greater reduction in the chaperone activity of HSP18 in the presence of 0.15 M and 0.5 M NaCl at other two higher temperatures (31°C and 43°C) (data not shown). Together, our data revealed that the interactions between HSP18 and chemically stressed client protein were weakened greatly in the presence of NaCl at higher temperature, which is the cause for reduction in the chaperone function of HSP18. Possibly, electrostatic interactions govern the recognition and binding of aggregation prone substrate proteins by HSP18 at normal as well as elevated temperatures.

**Fig 8:**
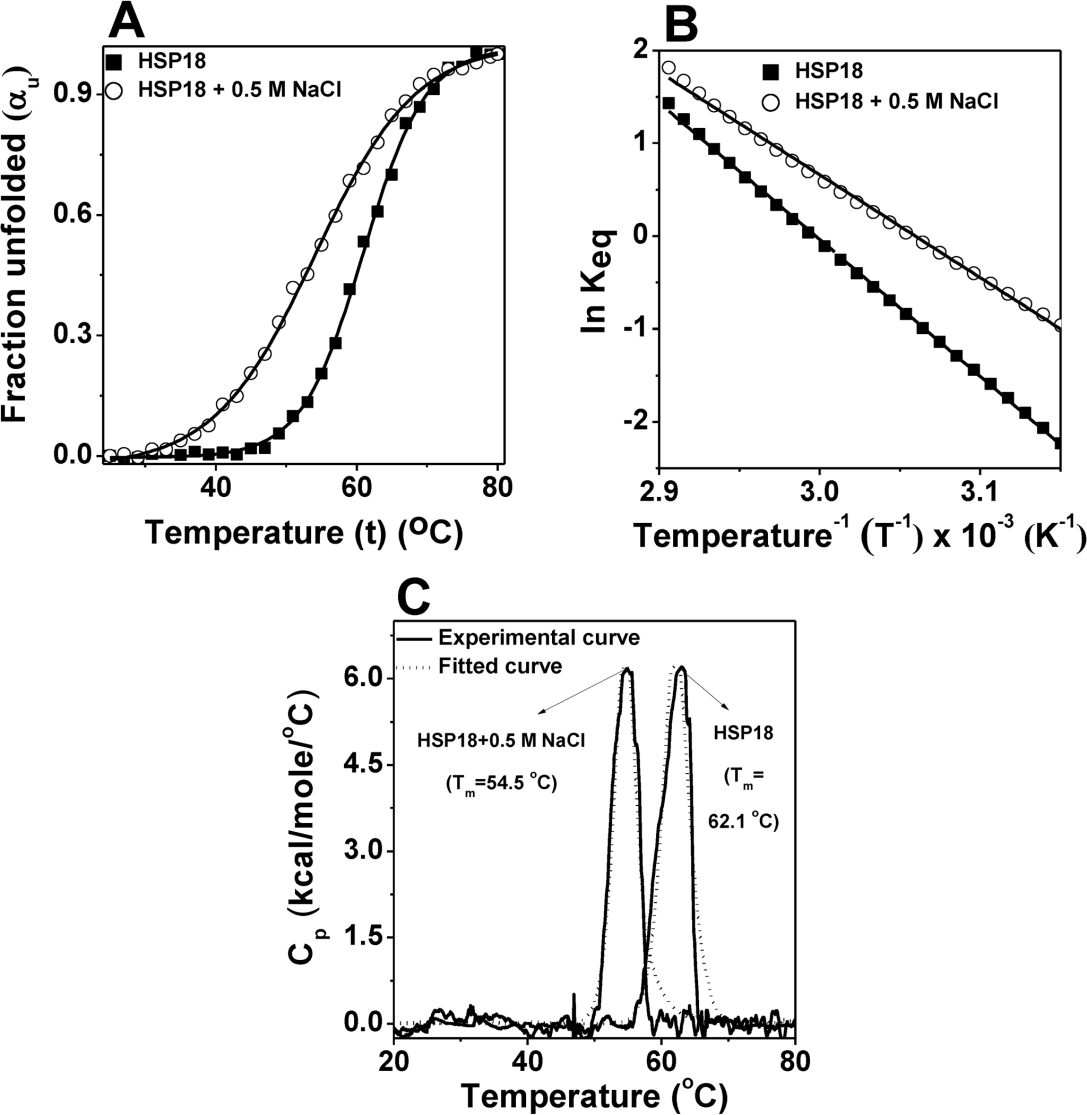
Effect of electrostatic interaction on the structural stability of HSP18. The thermal stability of HSP18 in the absence or presence of 0.5 M NaCl was determined using far-UV CD spectroscopy (panel A) and differential scanning calorimetry (panel C). The experimental data points (mentioned by symbols in panel A and sold lines in panel C) were fitted according to two-state model and the solid lines (in panel A) and dotted lines (in panel C) represent the best fit. **(B)** The data represented in panel A was fitted to Eq 5 in order to obtain the values of van’t Hoff enthalpy (∆H_vH_) HSP18 in the absence or presence of 0.5 M NaCl.

Once it was concluded that electrostatic interactions are important for the chaperone function of *M*. *leprae* HSP18, we were keen to know the molecular basis for the decreased chaperone function of HSP18 when the electrostatic forces were weakened by addition of NaCl. Electrostatic interactions are noncovalent bonding interactions, either attractive or repulsive in nature. It includes coulombic interactions and more sequence specific interactions like ion pairing or hydrogen bonding [52]. Electrostatic interaction is known to play key role in protein stability and function [53, 54]. It has long been implicated in maintaining thermal stability of thermophilic proteins [55, 56]. Valle *et al*. reported that Ca^2+^ ion induced decreased thermal stability of α-crystallin caused mainly due to electrostatic interaction between Ca^2+^/α-crystallin which eventually led to its decreased chaperone functionality [57]. So, the structural stability of HSP18 in the absence and presence of 0.5 M NaCl was compared against thermal stress using far-UV CD measurements. The change in the ellipticity magnitude at 222 nm was monitored over a temperature range from 25°C to 80°C. Then, the fraction unfolded (α_U_) for both systems were calculated using the following equation:

$$\alpha_{U}=\frac{\theta_{F}-\theta_{T}}{\theta_{F}-\theta_{U}}$$ Eq 4

where θ_F_ represents ellipticity value measured at 25°C for the native protein, θ_t_ represents ellipticity value measured at any temperature and θ_U_ represents ellipticity value measured at 80°C for the unfolded protein. As shown in Fig 8A, the thermal denaturation profiles of both proteins (HSP18 in the absence and presence of 0.5 M NaCl) were sigmoidal in nature. Although formation of an intermediate state cannot be negated, a two state transition model exhibited a significantly good fitting. Sigmoidal analysis of far-UV CD profiles at 222 nm demonstrated that HSP18 in absence of any NaCl underwent thermal unfolding with a midpoint transition or melting temperature (T_m_) of 60.9°C (Table 3). In the presence of 0.5 M NaCl, the T_m_ value shifted to 54°C (Fig 8A and Table 3). The decrease in T_m_ value (~6.9°C) clearly suggested that the presence of NaCl significantly reduced the structural stability of HSP18. As thermal denaturation profiles of HSP18 in the absence and presence of 0.5 M NaCl gave a significantly good fit with the two state transition model, the change in enthalpy (∆H_vH_) of this thermal transition was evaluated using van’t Hoff equation:

$$\ln K_{eq(Folded\to Unfolded)}=\frac{-\Delta H_{vH}}{RT}+\frac{\Delta S}{R}$$ Eq 5

where K_eq_(folded→unfolded) = α_U_/(1 - α_U_). The value of van’t Hoff enthalpy (ΔH_vH_) associated with the thermal transition of HSP18 (in absence of NaCl) was ~122.5 kJ/mol, which decreased to 92.2 kJ/mol for HSP18 in the presence of 0.5 M NaCl (Fig 8B and Table 3) which further confirmed that weakening of electrostatic interactions lowered the structural stability of HSP18 under thermal stress. The reversibility of this unfolding process of HSP18 in the absence or presence of 0.5 M NaCl was also checked by reheating the protein sample immediately after cooling at 25°C from the previous scan. It is worthwhile to mention that the T_m_ and ΔH_vH_ values so obtained from the reheating process were similar to that of during first heating. Therefore, it was inferred that the unfolding of HSP18 in the absence and presence of 0.5 M NaCl is reversible in nature.

**Table 3:** Thermodynamic parameters for thermal unfolding of HSP18 in the absence and presence of 0.5 M NaCl obtained from far UV-CD and DSC measurements.

System studied	Using far UV-CD		Using differential scanning calorimetry		
	T_m_ (°C)	∆H_vH_ (kJ/mol)	T_m_ (°C)	∆H_cal_ (kJ/mol)	∆H_vH_ (kJ/mol)
HSP18	60.9 ± 0.3	122.5 ± 2.1	62.1 ± 0.2	135.1 ± 1.9	135.9 ± 1.7
HSP18+0.5 M NaCl	54.0 ± 0.4	92.2 ± 1.8	54.5 ± 0.2	120.3 ± 2.7	121.0 ± 1.6

The effect of NaCl on the structural stability of HSP18 was further assessed by differential scanning calorimetry (DSC). We also performed this experiment to understand the presence of any intermediate during the thermal unfolding of HSP18 in the absence and presence of 0.5 M NaCl. DSC experiments revealed sharp transition during the unfolding of HSP18 in the absence and presence of 0.5 M NaCl (Fig 8C). Also, alterations in the melting temperature (T_m_) and van't Hoff enthalpy changes (ΔH_vH_) were observed in the presence of 0.5 M NaCl (Fig 8C and Table 3). While HSP18 in absence of NaCl exhibited a T_m_ and ΔH_vH_ of ~62.1°C and ~ 135.9 kJ/mole, respectively, the magnitude of T_m_ and ΔH_vH_ associated with the unfolding of HSP18 in the presence of 0.5 M NaCl were reduced (~54.5°C and 121 kJ/mole, respectively). These results were in agreement with our thermal denaturation experiments which were carried out using far-UV CD spectroscopy (Fig 8A and Table 3). Moreover, the unfolding process in DSC measurements produced narrow as well as relatively symmetric peak for HSP18 in the absence or presence of 0.5 M NaCl (Fig 8C) which indicated that both unfolding transitions may be two-state, reversible and highly cooperative [58]. Further verification of two step behavior was done by comparing the calorimetric (ΔH_cal_) and van't Hoff enthalpy changes (ΔH_vH_) as obtained from DSC measurements. The ΔH_cal_ and ΔH_vH_ magnitudes were found to be almost equal for the unfolding of HSP18 in the absence and presence of 0.5 M NaCl (Table 3). These results further confirmed that the denaturation process of *M*. *leprae* HSP18 (in the absence and presence of 0.5 M NaCl) could be accurately approximated by a two step model and rules out the possibility for the existence of an intermediate state [59]. As in both the systems, the existence of only two states were confirmed i.e. native state and the fully denatured state, the denaturation process of HSP18 in the absence or presence of 0.5 M NaCl could be termed as cooperative unfolding process. Reversibility of both the denaturation process was also checked by scanning to the thermal transition maximum, cooling to 20°C and scanning again upto 80°C. The T_m_, ΔH_cal_ and ΔH_vH_ values so obtained from the rescan remained almost unchanged. Thus these results again revealed that the thermal denaturation process of *M*. *leprae* HSP18 in the absence or presence of 0.5 M NaCl is a reversible, cooperative unfolding process. Previous studies on various sHSPs suggested that structural stability modulated their chaperone function [29, 48, 60]. Moreover, some studies revealed that enhancement in the chaperone activity of various sHSPs are accompanied by increased structural stability [29, 48, 60]. Thus it can be inferred from these data that weakening of the electrostatic interactions led to a decrease in the structural stability of HSP18, which reduced the chaperone activity of this protein.

**Fig 9:**
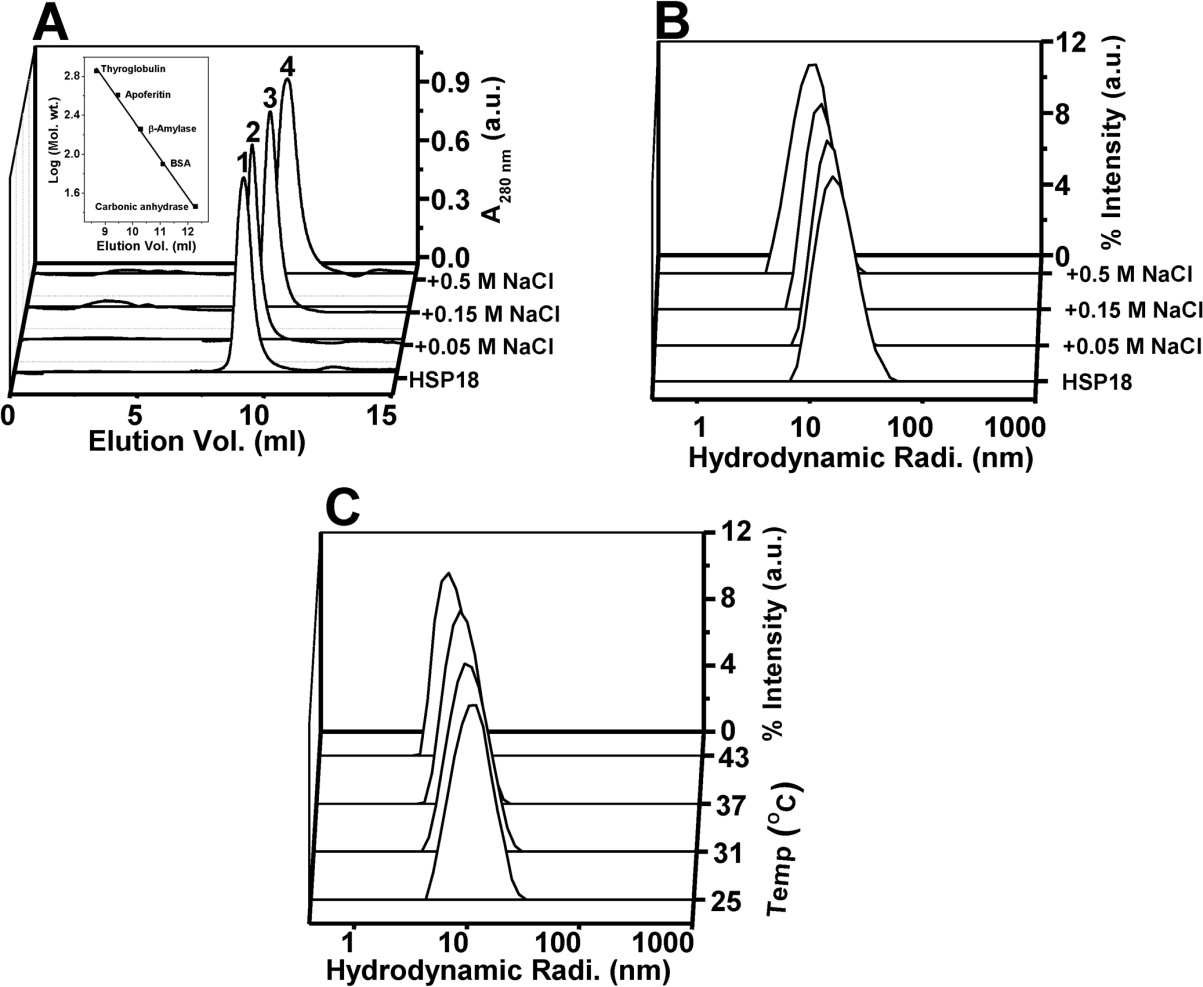
Effect of NaCl on the oligomeric mass/size of *M*. *leprae* HSP18. **(A)** Gel filtration profile of HSP18 in the absence or presence of 0.05–0.5 M NaCl at 25°C. TSK-GEL G4000SW_XL_ column (7.8 mm x 30 cm; 5 μm) was first equilibrated with 50 mM phosphate buffer (pH 7.5) with or without 0.05–0.5 M NaCl. Subsequently, 50 μl of HSP18, pre-incubated without or with 0.05–0.5 M NaCl was injected into the column. The flow rate used in this experiment was 0.5 ml/min. The oligomeric mass of different HSP18 samples was estimated using the standard curve (inset). **(B)** Intensity particle size distribution spectra of *M*. *leprae* HSP18 in the absence or presence of 0.05–0.5 M NaCl at 25°C. **(C)** Intensity particle size distribution spectra of *M*. *leprae* HSP18 in the presence of 0.5 M NaCl at different temperatures (25, 31, 37 and 43°C). Each spectrum is an average of 48 scans. HSP18 was incubated at respective temperatures in the absence or presence of 0.05–0.5 M NaCl for 1 hr prior to reading. Protein concentration used was 0.5 mg/ml in 50 mM phosphate buffer, pH 7.5.

To investigate whether the decreased structural stability of the protein due to the weakening of the electrostatic interactions, is accompanied by alteration in its static oligomeric assembly, the oligomaric size/mass of HSP18 was estimated in the absence and presence of 0.05–0.5 M NaCl at 25°C using gel filtration chromatography. The native HSP18 (in absence of NaCl) was eluted with a single peak at 9.05 ml (Fig 9A, peak 1). The peak position of HSP18 elution was shifted to 9.15 ml, 9.61 ml and 10.55 ml in the presence of 0.05 M, 0.15 M and 0.5 M NaCl, respectively (Fig 9A, peaks 2–4) which indicated the dissociation of static oligomeric assembly of HSP18 in the presence of NaCl. To quantify the oligomeric mass corresponding to these elution volumes, a standard curve was generated with several molecular weight standards (Fig 9A, *inset*). The oligomeric mass of HSP18 in absence of NaCl was found to be 563.6 kDa which is in well agreement with our previous estimation [29] In contrast, the oligomeric mass of HSP18 in the presence of 0.05 M, 0.15 M and 0.5 M NaCl was found to be 514, 335.5 and 223.7 kDa, respectively. Altogether, these data suggest that the electrostatic interactions led to the reduction in the oligomeric size of HSP18, consisting of 29 subunits in absence of NaCl to approximately 12 subunits in the presence of 0.5 M NaCl.

To confirm this fact, we also estimated the hydrodynamic radius of HSP18 in the absence or presence of 0.05–0.5 M NaCl using dynamic light scattering experiment at 25°C. This study clearly ascertained our gel filtration chromatographic results and showed that static oligomeric size of *M*. *leprae* HSP18 was reduced with increasing concentrations of NaCl (Fig 9B). In absence of NaCl, the observed hydrodynamic radius of *M*. *leprae* HSP18 was ~18.2 nm, which was reduced upto ~17.4 nm in the presence of 0.05 M NaCl. When NaCl concentrations were raised to 0.15 and 0.5 M, the observed hydrodynamic radius of M. *leprae* HSP18 was further reduced and found to be centered around ~16.5 and ~14.9 nm, respectively (Fig 9B). Thus a net decrease of ~3.3 nm in the hydrodynamic radius of *M*. *leprae* HSP18 was observed in the presence of 0.5 M NaCl at 25°C. We also explored how the static oligomeric size of HSP18 is altered in the presence of 0.5 M NaCl at higher temperatures (31, 37 and 43°C). As gel filtration chromatography is usually carried out at room temperature, and not at higher temperatures because of possible interaction between column matrix and protein, dynamic light scattering experiments were carried for this particular investigation. We observed that the magnitude of hydrodynamic radius of HSP18 decreased further with increasing temperatures (Fig 9C). While at 25°C, the hydrodynamic radius of HSP18 in the presence of 0.5 M NaCl was~14.9 nm, the same was found to be reduced to ~13.6 and ~12.8 nm at 31°C and 37°C respectively (Fig 9C). Further reduction in its hydrodynamic radius (~11.2 nm) was observed when the temperature was raised to 43°C, thereby resulting a net decrease of ~3.7 nm in the hydrodynamic radius of *M*. *leprae* HSP18 in the presence of 0.5 M NaCl (Fig 9C). This additional reduction of static oligomeric size of HSP18 at higher temperature in the presence of 0.5 M NaCl may be responsible for the further decrease in the chaperone function of 0.5 M NaCl incubated HSP18 (Figs 5 and 6). Previously, several studies demonstrated that reduced structural stability of sHSPs is often associated with dissociation of their static oligomeric assembly [61, 62]. These perturbations often reduced the chaperone function of these sHSPs [61, 62]. Recently, our group has also shown that a point mutation in the “α-crystallin domain” of *M*. *leprae* HSP18 caused its structural destabilization and dissociation of its oligomeric assembly [29]. The same study also revealed that such alteration in the quarternary structure and stability of the HSP18 was accompanied by reduced chaperone function. Therefore, we can conclude that weakening of electrostatic interactions in *M*. *leprae* HSP18 led to the oligomeric dissociation and structural destabilization which perhaps reduced its chaperone function. The reduced chaperone function of *M*. *leprae* HSP18 at elevated temperatures in the presence of NaCl was likely due to the reduction in its static oligomeric size, hinting over the fact that static oligomeric assembly is pre-reqisite for the chaperone function of *M*. *leprae* HSP18.

**Fig 10:**
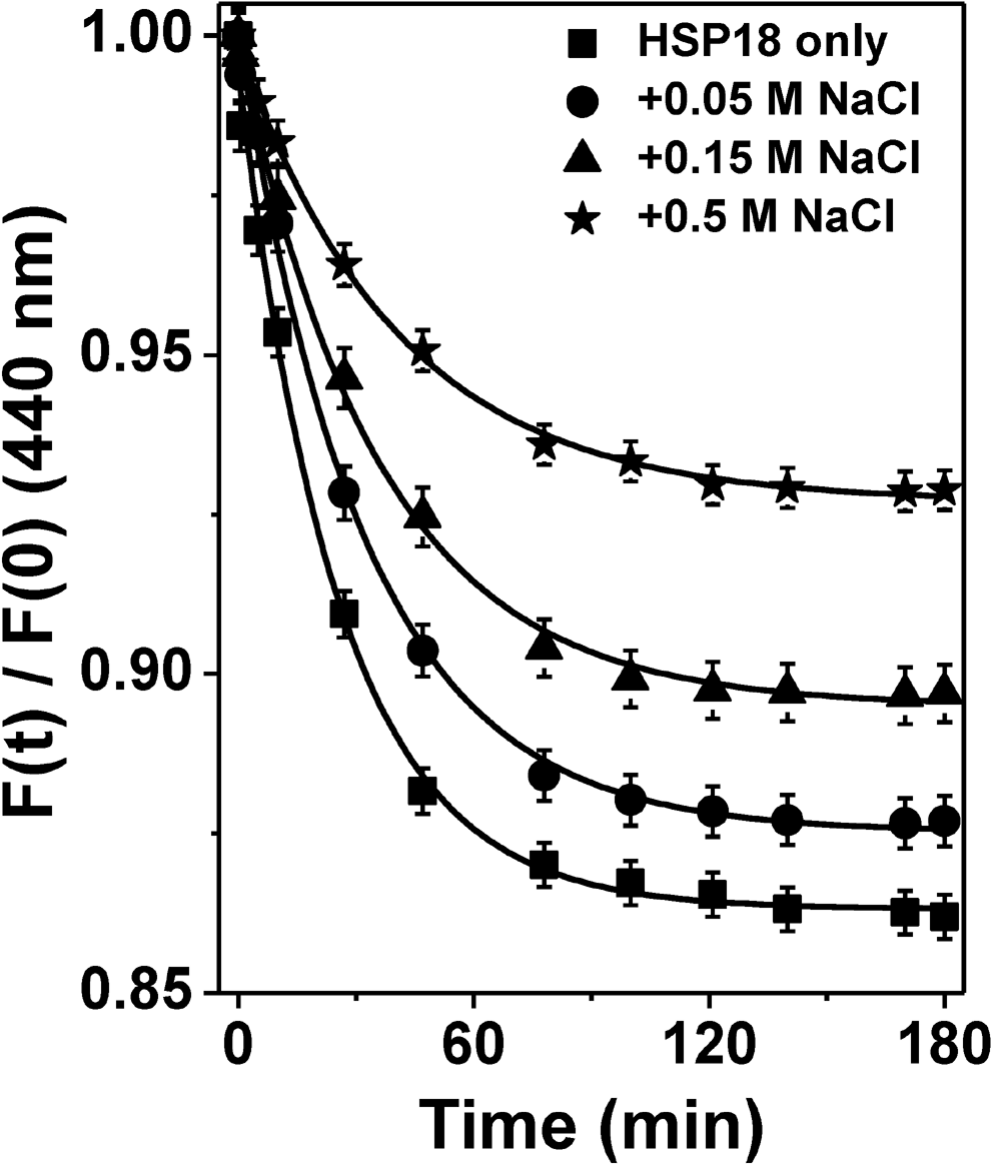
Effect of NaCl on subunit exchange rate of *M*. *leprae* HSP18. Subunit exchange between Alexa fluor-350 labeled and Alexa fluor-488 labeled HSP18 was monitored at 37°C in the absence or presence of 0.05–0.5 M NaCl. The concentration of both labeled protein was 1 mg/ml. The fluorescence spectra (400–600 nm) were recorded at various time intervals for different samples at 37°C. The excitation wavelength was 346 nm. In order to find out the subunit rate constant (k) of HSP18 in the absence or presence of 0.05–0.5 M NaCl, the curves were fitted according to the Eq 3.

Once it was evidenced that weakening of electrostatic interactions caused dissociation of static oligomeric assembly of HSP18, we were keenly interested to understand how the dynamics of oligomeric assembly of HSP18 are altered upon weakening of electrostatic interactions. We already demonstrated that the rate of subunit exchange is increased upon raising the temperature from 25°C to 43°C (Fig 3D) and may therefore play an important role in the enhancement of chaperone function of *M*. *leprae* HSP18 at elevated temperatures. We also demonstrated that weakening of electrostatic interactions resulted in the diminished chaperone activity of *M*. *leprae* HSP18 (Figs 5 and 6). Therefore, we hypothesized that kinetics of subunit exchange of HSP18 subunits may have retarded upon weakening of electrostatic interactions. To test this particular hypothesis, we executed the subunit exchange studies (by FRET) at different salt concentrations (0–0.5 M NaCl) at 37°C. In absence of NaCl, the rate constant for the subunit exchange of HSP18 subunit was 0.039 min^-1^ at 37°C (Fig 10, Table 4). In contrast, at the same temperature, the magnitude of the same was decreased in the presence of 0.05 M NaCl (to 0.031 min^-1^) (Fig 10, Table 4). Upon further increase in NaCl concentrations (0.15 and 0.5 M), the subunit exchange rate constant of *M*. *leprae* HSP18 subunits was found to be reduced further (0.028 min^-1^ and 0.024 min^-1^, respectively) (Fig 10, Table 4). The rate constant value of subunit exchange of HSP18 subunit was also reduced in the presence of 0.5 M NaCl at 25°C (S1 Table). Thus it is evident from here that dynamics of subunit exchange of *M*. *leprae* HSP18 is lowered when its electrostatic interactions are weakened. This leads to assume that the subunits of HSP18 interact with each other through electrostatic interactions and possibly the inter/intrapolypeptide interactions within the HSP oligomer affect/alter upon weakening of electrostatic interaction. Perhaps rearrangements of the subunits are essential for the binding of stressed client proteins and preventing their aggregation. Exhibition of similar trends in the subunit exchange phenomenon and chaperone function by *M*. *leprae* HSP18 clearly indicates that the dynamics of oligomeric assembly of *M*. *leprae* HSP18 are one of the determinants for the chaperone function of this protein at room temperature (25°C) as well as higher temperatures (31–43°C).

**Table 4:** Subunit exchange rate constant of *M*. *leprae* HSP18 in the absence or presence of 0.05–0.5 M NaCl at 37°C.

System studied	Subunit exchange rate constant (k) (min^-1^)
HSP18	0.039±0.002
HSP18 + 0.05 M NaCl	0.031±0.0014
HSP18 + 0.15 M NaCl	0.028±0.0011
HSP18 + 0.5 M NaCl	0.024±.001

**Fig 11:**
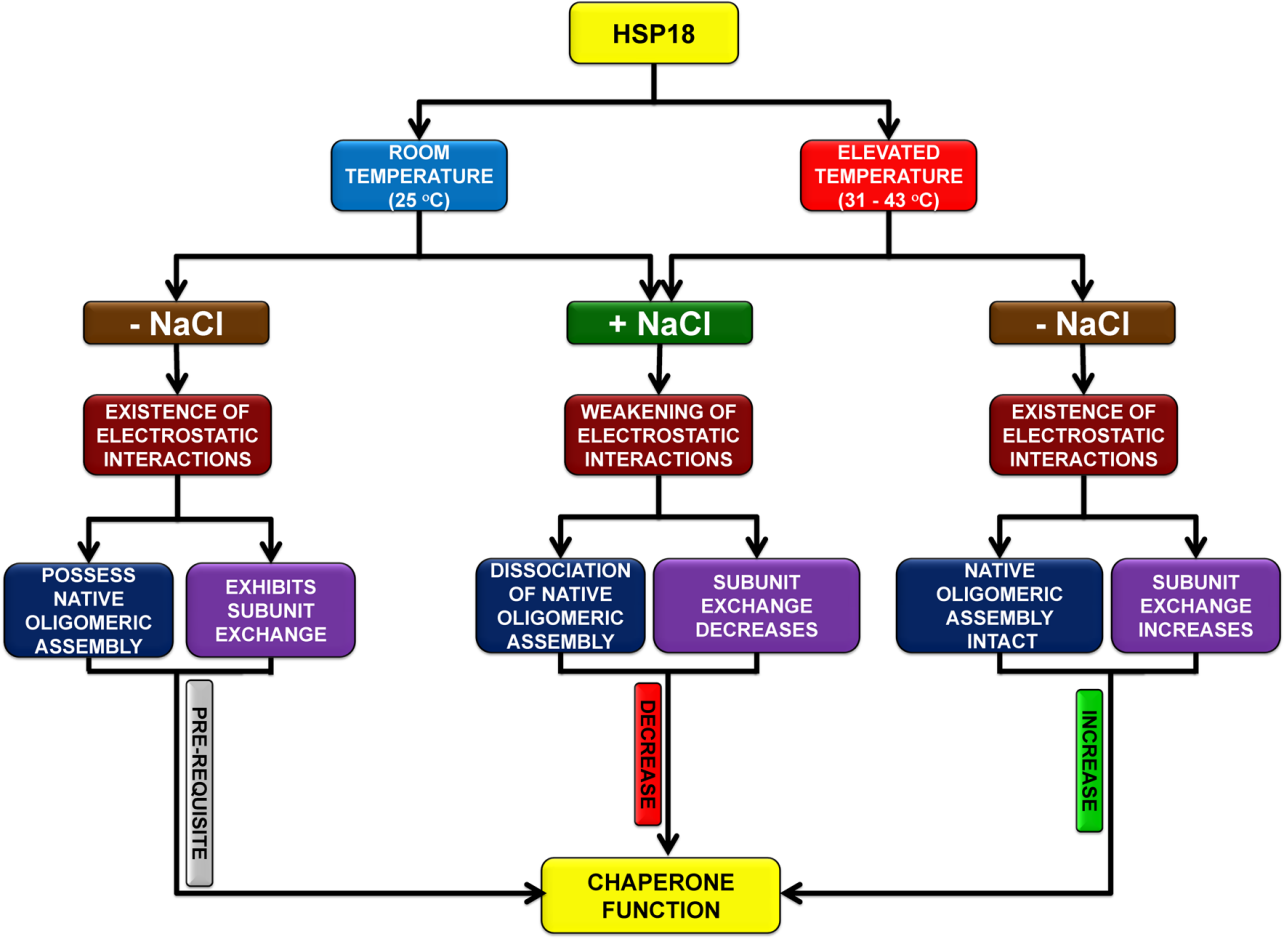
A proposed model which reflects the schematic mechanism for the chaperone function of *M*. *leprae* HSP18 at various temperatures.

In summary, the present study has demonstrated that HSP18 has a dynamic quaternary structure at room temperature (25°C) and apart from this unique feature, electrostatic interaction plays a crucial role in proper exhibition of chaperone function at this temperature. We also demonstrated that the subunit exchange rate of HSP18 subunits increases at different higher temperatures, resulting in increased chaperone function of HSP18. We successfully revealed that weakening of electrostatic interactions reduces the static oligomeric size, dynamics of oligomeric assembly and structural stability of HSP18. Altogether, these alterations resulted drastic decrease in its chaperone function at elevated temperatures. A possible model figure which reflects the schematic mechanism for the chaperone function of *M*. *leprae* HSP18 at various temperatures is shown in Fig 11. As HSP18 could prevent the insulin aggregation at physiological temperature (37°C) more efficiently, it implies that this protein can bind various aggregation prone client proteins *in vivo*. Previously, we demonstrated that HSP18 can prevent the thermal killing of *E*.*coli* at higher temperature [29]. Perhaps, enhanced chaperone activity of HSP18 at elevated temperature may be responsible for this prevention. Overall, the subunit exchange and electrostatic interactions play important role in the chaperone function of *M*. *leprae* HSP18. But, how the chaperone function of *M*. *leprae* HSP18 is modulated under various non thermal stresses such as hypoxia, nutrient depletion, oxidative stress etc., needs to be explored in details. These studies would improve our understanding on the molecular basis behind the chaperone function of *M*. *leprae* HSP18.

## Supporting Information

S1 Fig Effect of fluorophore labeling on the oligomeric mass of *M*. *leprae* HSP18 at 25°C. Gel filtration profile of HSP18 unlabelled or labelled with A-350/A-488 at 25°C. TSK-GEL G4000SW_XL_ column (7.8 mm x 30 cm, 5 μm) was equilibrated with 50 mM phosphate buffer, pH 7.5. Subsequently, 50 μL of HSP18, unlabelled/labelled with A-350/A-488 was injected into the column. Flow rate used was 0.5 ml/min. Profiles has been normalised to 0–1 scale.
(TIF)

S2 Fig Chaperone activity with varying concentration of HSP18 in the presence of 0.15 M NaCl at 25°C. Percentage protection by HSP18 against DTT-induced insulin aggregation at 25°C. The ratio of client protein to HSP18 (w/w) was 1:1.2, 1:1.5 and 1:1.8, respectively. Data are means ± standard deviation from triplicate determinations. *p< 0.05 and **p< 0.005.
(TIF)

S1 Table Subunit exchange rate constant of *M*. *leprae* HSP18 in the absence or presence of 0.5 M NaCl at 25°C. (DOCX)
