# Supplementary figures and images for: Role of Subunit Exchange and Electrostatic Interactions on the Chaperone Activity of Mycobacterium leprae HSP18
Source: PLoS One. 2015 Jun 22;10(6):e0129734. doi: 10.1371/journal.pone.0129734 (PMC4476693; doi:10.1371/journal.pone.0129734)

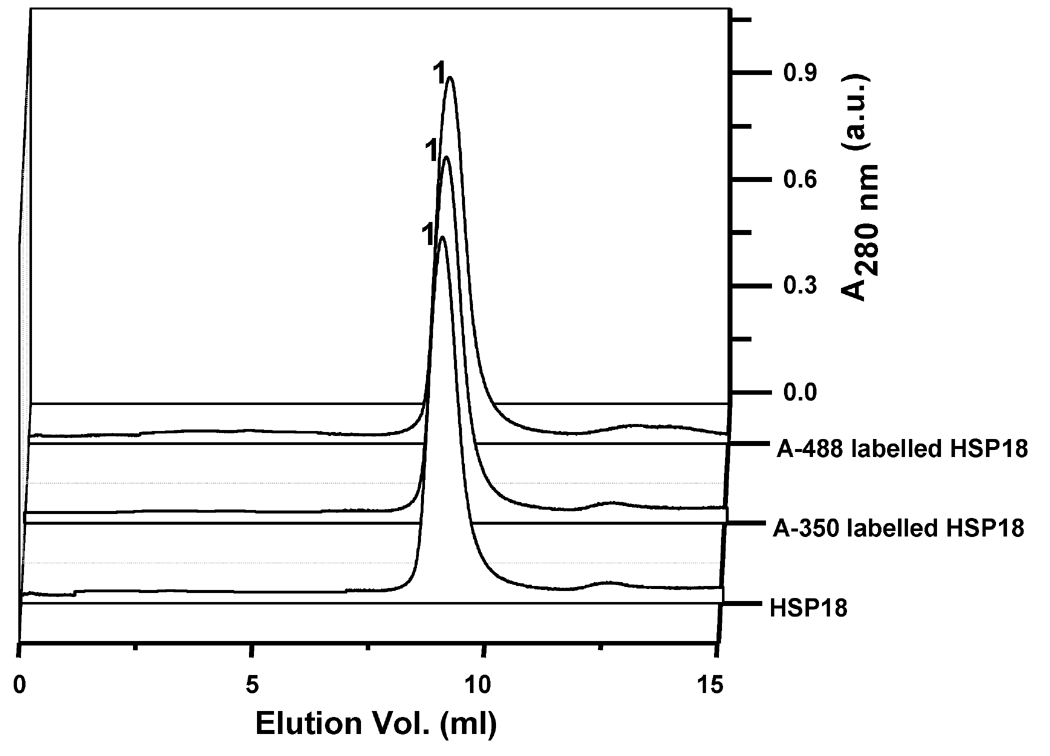

Supplement: S1 Fig — Gel filtration profile of HSP18 unlabelled or labelled with A-350/A-488 at 25°C. TSK-GEL G4000SWXL column (7.8 mm x 30 cm, 5 μm) was equilibrated with 50 mM phosphate buffer, pH 7.5. Subsequently, 50 μL of HSP18, unlabelled/labelled with A-350/A-488 was injected into the column. Flow rate used was 0.5 ml/min. Profiles has been normalised to 0–1 scale. (TIF) [file pone.0129734.s001.tif]

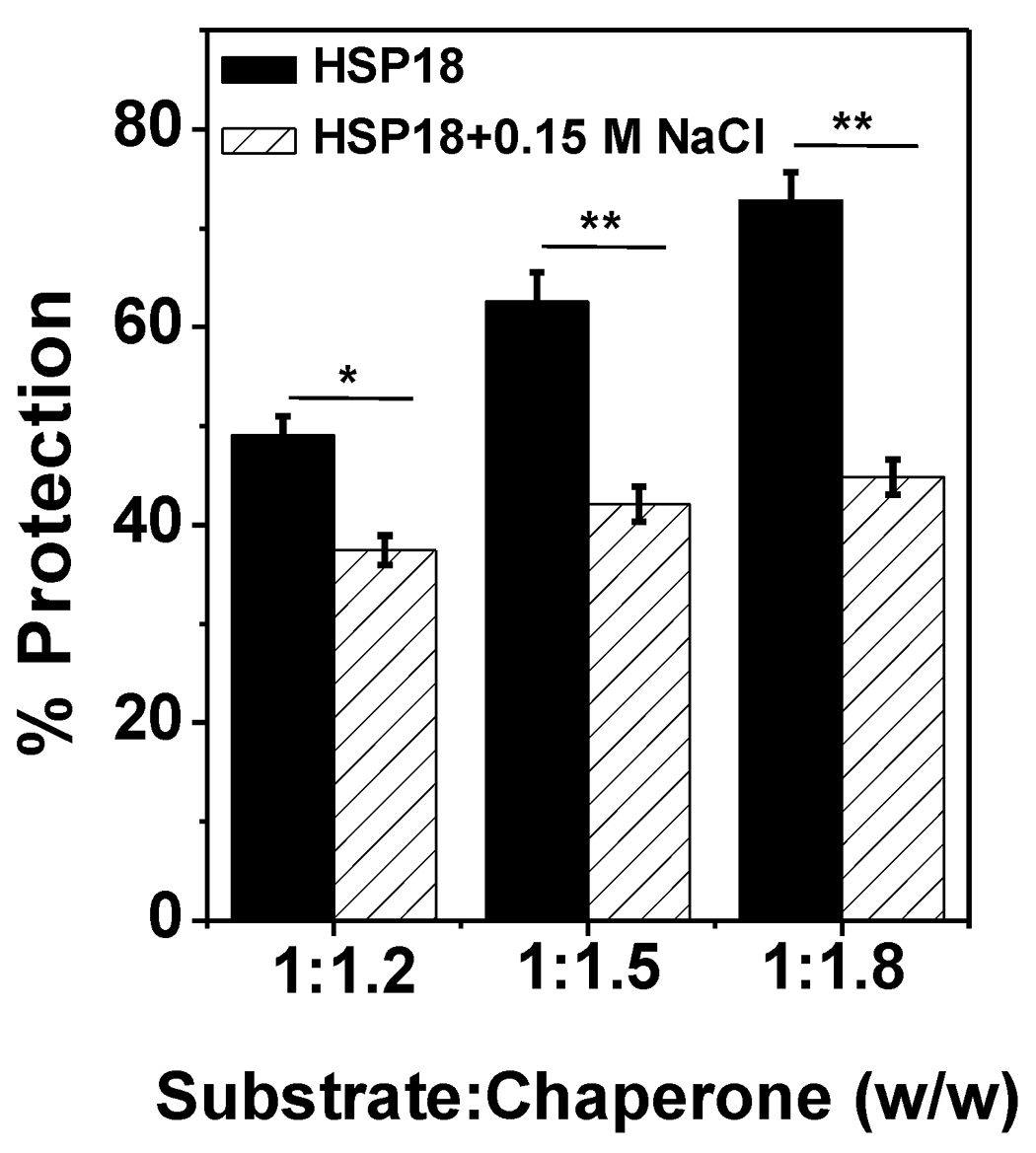

Supplement: S2 Fig — Percentage protection by HSP18 against DTT-induced insulin aggregation at 25°C. The ratio of client protein to HSP18 (w/w) was 1:1.2, 1:1.5 and 1:1.8, respectively. Data are means ± standard deviation from triplicate determinations. *p< 0.05 and **p< 0.005. (TIF) [file pone.0129734.s002.tif]
