# Supplementary material for: Role of Subunit Exchange and Electrostatic Interactions on the Chaperone Activity of Mycobacterium leprae HSP18
Source: PLoS One. 2015 Jun 22;10(6):e0129734. doi: 10.1371/journal.pone.0129734 (PMC4476693; doi:10.1371/journal.pone.0129734)
Supplement: S1 Table — (DOCX) [file pone.0129734.s003.docx]

**S1 Table. Subunit exchange rate constant of *M. leprae* HSP18 in absence or presence of 0.5 M NaCl at 25 °C.**

| **System studied** | **Subunit exchange rate constant (k) (min^-1^)** |
| --- | --- |
| HSP18 | 0.019 ± 0.004 |
| HSP18 + 0.5 M NaCl | 0.014 ± 0.001 |
